# Supplementary material for: Does increasing biodiversity in an urban woodland setting promote positive emotional responses in humans? A stress recovery experiment using 360-degree videos of an urban woodland
Source: PLoS One. 2024 Feb 7;19(2):e0297179. doi: 10.1371/journal.pone.0297179 (PMC10849218; doi:10.1371/journal.pone.0297179)
Supplement: S1 Appendix — Species lists, codes description and perceived biodiversity crosstabulation. (DOCX) [file pone.0297179.s001.docx]

Supporting Information

**Table S1.** Plant taxa surveyed in the urban woodland study area

| **Species** | **Distance from the camera** |
| --- | --- |
| *Acer campestre* | Far > 5m |
| *Acer sp.* | Close < 5m |
| *Aesculus hippocastanum* | Far > 5m |
| *Anthriscus sylvestris* | Far > 5m |
| *Berberis sp.* | Close < 5m |
| *Bluddleja davidii* | Far > 5m |
| *Carex sp.* | Close < 5m |
| *Corylus avellana* | Close < 5m |
| *Crataegus monogyna* | Close < 5m |
| *Fagus sylvatica* | Close < 5m |
| *Geum urbanum* | Close < 5m |
| *Hedera helix* | Close < 5m |
| *Hyacinthoides hyspanica* | Far > 5m |
| *Hyacinthoides non-scripta* | Close < 5m |
| *Ilex aquifolium* | Close < 5m |
| *Leycesteria formosa* | Far > 5m |
| *Lonicera nitida* | Far > 5m |
| *Malus sylvestris* | Far > 5m |
| *Myosotis arvensis* | Far > 5m |
| *Narcissus sp.* | Far > 5m |
| *Pinus strobus* | Far > 5m |
| *Pinus sylvestris* | Close < 5m |
| *Platanus hybrida* | Close < 5m |
| *Polygonatum multiflorum* | Far > 5m |
| *Prunus avium* | Far > 5m |
| *Prunus laurocerasus* | Close < 5m |
| *Pteridium aquilinum* | Close < 5m |
| *Quercus robur* | Close < 5m |
| *Ranunculus sp.* | Far > 5m |
| *Reynoutria japonica* | Far > 5m |
| *Rhododendron sp.* | Close < 5m |
| *Rubus spectabilis* | Close < 5m |
| *Rumex sp.* | Far > 5m |
| *Taraxacum sp.* | Far > 5m |
| *Taxus baccata* | Close < 5m |
| *Urtica dioca* | Far > 5m |

**Table S2.** Bird taxa – calls and songs that could be heard in condition 4

| **Species** |
| --- |
| *Columba palumbus* |
| *Cyanistes caeruleus* |
| *Erithacus rubecula* |
| *Phylloscopus collybita* |
| *Troglodytes troglodytes* |
| *Turdus merula* |

**Table S3**. Content analysis results. What did the participants notice to evaluate the environment as “good” or “very good”?

|  |  |  | **Condition** | | | |
| --- | --- | --- | --- | --- | --- | --- |
| **Theme** | **Description** | **Count** | **v1** | **v2** | **v3** | **v4** |
| **Sounds** | Ambient sounds (wind, water, birds) | 55 | 8 | 12 | 12 | **24** |
| **Flowers** | Flowers on the ground, around the camera. These are the flowers used to increase the biodiversity count. | 35 | 0 | 14 | 11 | 10 |
| **Trees** | Trees mentioned explicitly, often commenting on their abundance, health, size. | 34 | 8 | 10 | 7 | 9 |
| Greenery | Abundance of the colour green, used the term "greenery" or collective terms such as "vegetation", "flora" but did not use more specific terms. | 33 | 10 | 5 | 9 | 9 |
| Diversity | Used the words "Diversity" and "Variety" to describe the environment. Also includes sentences such as "a lot of plants", "a rage of plants", "Many different plants", "many habitats". | 33 | 5 | 4 | 9 | **15** |
| General appreciation | General positive feeling of environment as a whole. Most of the comments mentioned the environment being "peaceful" | 31 | 11 | 7 | 8 | 5 |
| Human presence/absence | Human activity (clearing, logging, vehicle noise) or the absence of human disturbance (e.g. no rubbish) | 24 | 6 | 2 | 7 | 9 |
| Movement | Movement in the environment, such as the branches moving in the breeze or some insects flying close to the camera | 18 | 7 | 4 | 3 | 4 |
| Nature/Natural | Used the word "Natural" in general terms to describe the environment. Also includes sentences such as "full of Nature" | 17 | 8 | 5 | 2 | 2 |
| Not pertinent | The participant used this space to provide different kinds of feedbacks. | 15 | 2 | 3 | 4 | 6 |
| Unseen | Absence of something they would have expected to see. Such expectation derived from hearing (or believing to have heard) specific sounds in the ambience, such as water flowing | 12 | 5 | 4 | 1 | 2 |
| Forest/Woods | Words such as "forest" or "woodland" used to broadly describe the environment without mentioning anything more specific (e.g. trees) | 12 | 5 | 0 | 3 | 4 |
| Calm/Calming | The participant described the video as relaxing, calming, usually referring to the sounds | 11 | 4 | 2 | 1 | 4 |
| Bird box | Noticed the presence of a bird box on one of the trees. | 10 | 1 | 2 | 5 | 2 |
| Immersive | Used the words "immersive" or sentences like "it was like to be there" | 7 | 2 | 1 | 2 | 2 |
| Unnatural | Participants from conditions 3 and 4, mentioned that the experimental planting looked "not too artificial" or "out of place" | 7 | 0 | 0 | 4 | 3 |
| Insects | Presence of insects flying around the camera | 7 | 4 | 0 | 1 | 2 |
| Sky and lighting | Sky among the treetops and the light reaching the ground. | 7 | 0 | 4 | 1 | 2 |
| Technical value | Appreciation for the technical quality of the video. | 6 | 2 | 2 | 2 | 0 |
| Silence | The participant used the words "quiet" or "silence" to evaluate the environment | 5 | 2 | 0 | 3 | 0 |
| Safe | The participant rated the area safe for the development of plants and wildlife | 2 | 2 | 0 | 0 | 0 |
| Technical issue | Technical issues with the survey. | 2 | 1 | 0 | 1 | 0 |

**Table S4** Percentage of perceived biodiversity per each video condition

| **Percent Distribution of perceived biodiversity by group** | | | | |
| --- | --- | --- | --- | --- |
|  | **Condition video** | | | |
| **Perceived Biodiversity** | Control | plus 4 species | plus 21 species | plus 21 species and audio |
| Very bad | 0.0 | 0.0 | 2.2 | 2.2 |
| Bad | 5.4 | 6.0 | 5.4 | 5.5 |
| Neither good nor bad | 21.7 | 24.2 | 19.6 | 20.0 |
| Good | 50.0 | 55.6 | 46.7 | 47.7 |
| Very good | 22.8 | 25.4 | 26.1 | 26.6 |
