# Supplementary material for: Does increasing biodiversity in an urban woodland setting promote positive emotional responses in humans? A stress recovery experiment using 360-degree videos of an urban woodland
Source: PLoS One. 2024 Feb 7;19(2):e0297179. doi: 10.1371/journal.pone.0297179 (PMC10849218; doi:10.1371/journal.pone.0297179)
Supplement: S1 Dataset — (ZIP) [file pone.0297179.s002.zip › Dataset and statistics output/1 - Descriptive data.docx]

Descriptive data

**Frequencies**

| **Notes** | | |
| --- | --- | --- |
| Output Created | | 29-MAR-2023 12:10:03 |
| Comments | |  |
| Input | Active Dataset | DataSet1 |
|  | Filter | <none> |
|  | Weight | <none> |
|  | Split File | <none> |
|  | N of Rows in Working Data File | 372 |
| Missing Value Handling | Definition of Missing | User-defined missing values are treated as missing. |
|  | Cases Used | Statistics are based on all cases with valid data. |
| Syntax | | FREQUENCIES VARIABLES=gender age ethnicit first_ti device child_ou ins_1 perc_bio /ORDER=ANALYSIS. |
| Resources | Processor Time | 00:00:00.02 |
|  | Elapsed Time | 00:00:00.02 |

[DataSet1]

| **Statistics** | | | | | | | | | |
| --- | --- | --- | --- | --- | --- | --- | --- | --- | --- |
|  | | Question: What is your gender? | Question: How old are you? (age group) | Question: What is your ethnic group? | Question: Have you ever watched a 360-degree video before? (maybe on YouTube or Facebook) | Question: Which kind of device are you using to take this survey? | Question: How much time did you spend outdoor as a child? | Value of the Inclusion of Nature in Self scale | Perceptions of biodiversity reported by the participant after watching the video. |
| N | Valid | 372 | 372 | 365 | 372 | 372 | 372 | 372 | 372 |
|  | Missing | 0 | 0 | 7 | 0 | 0 | 0 | 0 | 0 |

**Frequency Table**

**Gender**

| **Question: What is your gender?** | | | | | |
| --- | --- | --- | --- | --- | --- |
|  | | Frequency | Percent | Valid Percent | Cumulative Percent |
| Valid | Male | 127 | 34.1 | 34.1 | 34.1 |
|  | Female | 236 | 63.4 | 63.4 | 97.6 |
|  | Non-binary / third gender | 5 | 1.3 | 1.3 | 98.9 |
|  | Prefer not to say | 4 | 1.1 | 1.1 | 100.0 |
|  | Total | 372 | 100.0 | 100.0 |  |

**Age**

| **Question: How old are you? (age group)** | | | | | |
| --- | --- | --- | --- | --- | --- |
|  | | Frequency | Percent | Valid Percent | Cumulative Percent |
| Valid | 18 - 24 | 163 | 43.8 | 43.8 | 43.8 |
|  | 25 - 34 | 129 | 34.7 | 34.7 | 78.5 |
|  | 35 - 44 | 38 | 10.2 | 10.2 | 88.7 |
|  | 45 - 54 | 20 | 5.4 | 5.4 | 94.1 |
|  | 55 - 64 | 8 | 2.2 | 2.2 | 96.2 |
|  | 65 - 74 | 13 | 3.5 | 3.5 | 99.7 |
|  | 75 - 84 | 1 | .3 | .3 | 100.0 |
|  | Total | 372 | 100.0 | 100.0 |  |

**Ethinicity**

| **Question: What is your ethnic group?** | | | | | |
| --- | --- | --- | --- | --- | --- |
|  | | Frequency | Percent | Valid Percent | Cumulative Percent |
| Valid | White (including English/Welsh/Scottish/Northern Irish/British, Irish, Gypsy or Irish Traveller and Any other White back | 242 | 65.1 | 66.3 | 66.3 |
|  | Mixed/Multiple ethnic groups | 31 | 8.3 | 8.5 | 74.8 |
|  | Asian/Asian British (Including Indian, Pakistani, Bangladeshi, Chinese and any other Asian background) | 70 | 18.8 | 19.2 | 94.0 |
|  | Black/African/Caribbean/Black British | 12 | 3.2 | 3.3 | 97.3 |
|  | Other ethnic group (please describe) | 10 | 2.7 | 2.7 | 100.0 |
|  | Total | 365 | 98.1 | 100.0 |  |
| Missing | System | 7 | 1.9 |  |  |
| Total | | 372 | 100.0 |  |  |

| **Question: Have you ever watched a 360-degree video before? (maybe on YouTube or Facebook)** | | | | | |
| --- | --- | --- | --- | --- | --- |
|  | | Frequency | Percent | Valid Percent | Cumulative Percent |
| Valid | Yes | 259 | 69.6 | 69.6 | 69.6 |
|  | No | 113 | 30.4 | 30.4 | 100.0 |
|  | Total | 372 | 100.0 | 100.0 |  |

| **Question: Which kind of device are you using to take this survey?** | | | | | |
| --- | --- | --- | --- | --- | --- |
|  | | Frequency | Percent | Valid Percent | Cumulative Percent |
| Valid | Desktop | 87 | 23.4 | 23.4 | 23.4 |
|  | Laptop | 240 | 64.5 | 64.5 | 87.9 |
|  | Smartphone | 40 | 10.8 | 10.8 | 98.7 |
|  | Tablet | 5 | 1.3 | 1.3 | 100.0 |
|  | Total | 372 | 100.0 | 100.0 |  |

| **Question: How much time did you spend outdoor as a child?** | | | | | |
| --- | --- | --- | --- | --- | --- |
|  | | Frequency | Percent | Valid Percent | Cumulative Percent |
| Valid | A lot | 178 | 47.8 | 47.8 | 47.8 |
|  | A moderate amount | 155 | 41.7 | 41.7 | 89.5 |
|  | A little | 37 | 9.9 | 9.9 | 99.5 |
|  | None at all | 2 | .5 | .5 | 100.0 |
|  | Total | 372 | 100.0 | 100.0 |  |

| **Value of the Inclusion of Nature in Self scale** | | | | | |
| --- | --- | --- | --- | --- | --- |
|  | | Frequency | Percent | Valid Percent | Cumulative Percent |
| Valid | 1.00 | 20 | 5.4 | 5.4 | 5.4 |
|  | 2.00 | 71 | 19.1 | 19.1 | 24.5 |
|  | 3.00 | 54 | 14.5 | 14.5 | 39.0 |
|  | 4.00 | 43 | 11.6 | 11.6 | 50.5 |
|  | 5.00 | 103 | 27.7 | 27.7 | 78.2 |
|  | 6.00 | 70 | 18.8 | 18.8 | 97.0 |
|  | 7.00 | 11 | 3.0 | 3.0 | 100.0 |
|  | Total | 372 | 100.0 | 100.0 |  |

| **Perceptions of biodiversity reported by the participant after watching the video.** | | | | | |
| --- | --- | --- | --- | --- | --- |
|  | | Frequency | Percent | Valid Percent | Cumulative Percent |
| Valid | Very bad | 4 | 1.1 | 1.1 | 1.1 |
|  | Bad | 15 | 4.0 | 4.0 | 5.1 |
|  | Neither good nor bad | 69 | 18.5 | 18.5 | 23.7 |
|  | Good | 197 | 53.0 | 53.0 | 76.6 |
|  | Very good | 87 | 23.4 | 23.4 | 100.0 |
|  | Total | 372 | 100.0 | 100.0 |  |
