# Supplementary material for: Does increasing biodiversity in an urban woodland setting promote positive emotional responses in humans? A stress recovery experiment using 360-degree videos of an urban woodland
Source: PLoS One. 2024 Feb 7;19(2):e0297179. doi: 10.1371/journal.pone.0297179 (PMC10849218; doi:10.1371/journal.pone.0297179)
Supplement: S1 Dataset — (ZIP) [file pone.0297179.s002.zip › Dataset and statistics output/2 - Affective responses to the biodiversity in the videos.docx]

Affective responses to the biodiversity in the video

**Negative affect**

**General Linear Model**

| **Notes** | | |
| --- | --- | --- |
| Output Created | | 29-MAR-2023 13:30:03 |
| Comments | |  |
| Input | Active Dataset | DataSet1 |
|  | Filter | <none> |
|  | Weight | <none> |
|  | Split File | <none> |
|  | N of Rows in Working Data File | 372 |
| Missing Value Handling | Definition of Missing | User-defined missing values are treated as missing. |
|  | Cases Used | Statistics are based on all cases with valid data for all variables in the model. |
| Syntax | | GLM na_t1 na_t2 na_t3 BY condition /WSFACTOR=Stage 3 Simple(1) /MEASURE=Negative_affect /METHOD=SSTYPE(3) /EMMEANS=TABLES(Stage) COMPARE ADJ(BONFERRONI) /PRINT=DESCRIPTIVE ETASQ OPOWER /CRITERIA=ALPHA(.05) /WSDESIGN=Stage /DESIGN=condition. |
| Resources | Processor Time | 00:00:00.00 |
|  | Elapsed Time | 00:00:00.02 |

| **Within-Subjects Factors** | |
| --- | --- |
| Measure: Negative_affect | |
| Stage | Dependent Variable |
| 1 | na_t1 |
| 2 | na_t2 |
| 3 | na_t3 |

| **Between-Subjects Factors** | | | |
| --- | --- | --- | --- |
|  | | Value Label | N |
| The experimental conditionn to which each participant was assigned | 1 | Control | 92 |
|  | 2 | plus 4 species | 90 |
|  | 3 | plus 21 species | 92 |
|  | 4 | plus 21 species and audio | 98 |

| **Descriptive Statistics** | | | | |
| --- | --- | --- | --- | --- |
|  | The experimental conditionn to which each participant was assigned | Mean | Std. Deviation | N |
| Negative Affect baseline score | Control | 7.2935 | 3.28680 | 92 |
|  | plus 4 species | 7.8667 | 3.30543 | 90 |
|  | plus 21 species | 8.3478 | 4.19428 | 92 |
|  | plus 21 species and audio | 7.4796 | 3.20190 | 98 |
|  | Total | 7.7419 | 3.52550 | 372 |
| Negative Affect score after stressor | Control | 9.0217 | 3.97237 | 92 |
|  | plus 4 species | 9.3667 | 4.22526 | 90 |
|  | plus 21 species | 10.1848 | 4.52327 | 92 |
|  | plus 21 species and audio | 9.3980 | 4.10366 | 98 |
|  | Total | 9.4919 | 4.21363 | 372 |
| Negative Affect score after video | Control | 6.5652 | 3.00660 | 92 |
|  | plus 4 species | 6.4444 | 2.50443 | 90 |
|  | plus 21 species | 6.8587 | 3.75801 | 92 |
|  | plus 21 species and audio | 6.7551 | 3.00193 | 98 |
|  | Total | 6.6586 | 3.09318 | 372 |

| **Multivariate Tests**^a^ | | | | | | | | |
| --- | --- | --- | --- | --- | --- | --- | --- | --- |
| Pillai's Trace | | | | | | | | |
| Effect | Value | F | Hypothesis df | Error df | Sig. | Partial Eta Squared | Noncent. Parameter | Observed Power^d^ |
| Stage | .385 | 115.048^b^ | 2.000 | 367.000 | <.001 | .385 | 230.097 | 1.000 |
| Stage * condition | .021 | 1.301 | 6.000 | 736.000 | .254 | .010 | 7.804 | .515 |
| a. Design: Intercept + condition Within Subjects Design: Stage | | | | | | | | |
| b. Exact statistic | | | | | | | | |
| d. Computed using alpha = .05 | | | | | | | | |

| **Mauchly's Test of Sphericity**^a^ | | | | | | | |
| --- | --- | --- | --- | --- | --- | --- | --- |
| Measure: Negative_affect | | | | | | | |
| Within Subjects Effect | Mauchly's W | Approx. Chi-Square | df | Sig. | Epsilon^b^ | | |
|  |  |  |  |  | Greenhouse-Geisser | Huynh-Feldt | Lower-bound |
| Stage | .885 | 44.704 | 2 | <.001 | .897 | .909 | .500 |
| Tests the null hypothesis that the error covariance matrix of the orthonormalized transformed dependent variables is proportional to an identity matrix. | | | | | | | |
| a. Design: Intercept + condition Within Subjects Design: Stage | | | | | | | |
| b. May be used to adjust the degrees of freedom for the averaged tests of significance. Corrected tests are displayed in the Tests of Within-Subjects Effects table. | | | | | | | |

| **Tests of Within-Subjects Effects** | | | | | | | | | |
| --- | --- | --- | --- | --- | --- | --- | --- | --- | --- |
| Measure: Negative_affect | | | | | | | | | |
| Source | | Type III Sum of Squares | df | Mean Square | F | Sig. | Partial Eta Squared | Noncent. Parameter | Observed Power^a^ |
| Stage | Sphericity Assumed | 1521.999 | 2 | 760.999 | 141.637 | <.001 | .278 | 283.274 | 1.000 |
|  | Greenhouse-Geisser | 1521.999 | 1.794 | 848.273 | 141.637 | <.001 | .278 | 254.130 | 1.000 |
|  | Huynh-Feldt | 1521.999 | 1.817 | 837.570 | 141.637 | <.001 | .278 | 257.377 | 1.000 |
|  | Lower-bound | 1521.999 | 1.000 | 1521.999 | 141.637 | <.001 | .278 | 141.637 | 1.000 |
| Stage * condition | Sphericity Assumed | 32.838 | 6 | 5.473 | 1.019 | .412 | .008 | 6.112 | .407 |
|  | Greenhouse-Geisser | 32.838 | 5.383 | 6.101 | 1.019 | .408 | .008 | 5.483 | .382 |
|  | Huynh-Feldt | 32.838 | 5.451 | 6.024 | 1.019 | .409 | .008 | 5.553 | .385 |
|  | Lower-bound | 32.838 | 3.000 | 10.946 | 1.019 | .384 | .008 | 3.056 | .277 |
| Error(Stage) | Sphericity Assumed | 3954.440 | 736 | 5.373 |  |  |  |  |  |
|  | Greenhouse-Geisser | 3954.440 | 660.277 | 5.989 |  |  |  |  |  |
|  | Huynh-Feldt | 3954.440 | 668.715 | 5.913 |  |  |  |  |  |
|  | Lower-bound | 3954.440 | 368.000 | 10.746 |  |  |  |  |  |
| a. Computed using alpha = .05 | | | | | | | | | |

| **Tests of Within-Subjects Contrasts** | | | | | | | | | |
| --- | --- | --- | --- | --- | --- | --- | --- | --- | --- |
| Measure: Negative_affect | | | | | | | | | |
| Source | Stage | Type III Sum of Squares | df | Mean Square | F | Sig. | Partial Eta Squared | Noncent. Parameter | Observed Power^a^ |
| Stage | Level 2 vs. Level 1 | 1132.766 | 1 | 1132.766 | 94.598 | <.001 | .204 | 94.598 | 1.000 |
|  | Level 3 vs. Level 1 | 442.357 | 1 | 442.357 | 61.739 | <.001 | .144 | 61.739 | 1.000 |
| Stage * condition | Level 2 vs. Level 1 | 9.142 | 3 | 3.047 | .254 | .858 | .002 | .763 | .098 |
|  | Level 3 vs. Level 1 | 49.704 | 3 | 16.568 | 2.312 | .076 | .019 | 6.937 | .580 |
| Error(Stage) | Level 2 vs. Level 1 | 4406.608 | 368 | 11.974 |  |  |  |  |  |
|  | Level 3 vs. Level 1 | 2636.712 | 368 | 7.165 |  |  |  |  |  |
| a. Computed using alpha = .05 | | | | | | | | | |

| **Tests of Between-Subjects Effects** | | | | | | | | |
| --- | --- | --- | --- | --- | --- | --- | --- | --- |
| Measure: Negative_affect | | | | | | | | |
| Transformed Variable: Average | | | | | | | | |
| Source | Type III Sum of Squares | df | Mean Square | F | Sig. | Partial Eta Squared | Noncent. Parameter | Observed Power^a^ |
| Intercept | 23577.323 | 1 | 23577.323 | 2442.541 | <.001 | .869 | 2442.541 | 1.000 |
| condition | 34.630 | 3 | 11.543 | 1.196 | .311 | .010 | 3.588 | .321 |
| Error | 3552.225 | 368 | 9.653 |  |  |  |  |  |
| a. Computed using alpha = .05 | | | | | | | | |

**Estimated Marginal Means**

**Stage**

| **Estimates** | | | | |
| --- | --- | --- | --- | --- |
| Measure: Negative_affect | | | | |
| Stage | Mean | Std. Error | 95% Confidence Interval | |
|  |  |  | Lower Bound | Upper Bound |
| 1 | 7.747 | .182 | 7.388 | 8.106 |
| 2 | 9.493 | .218 | 9.063 | 9.922 |
| 3 | 6.656 | .161 | 6.339 | 6.972 |

| **Pairwise Comparisons** | | | | | | |
| --- | --- | --- | --- | --- | --- | --- |
| Measure: Negative_affect | | | | | | |
| (I) Stage | (J) Stage | Mean Difference (I-J) | Std. Error | Sig.^b^ | 95% Confidence Interval for Difference^b^ | |
|  |  |  |  |  | Lower Bound | Upper Bound |
| 1 | 2 | -1.746^*^ | .180 | <.001 | -2.178 | -1.314 |
|  | 3 | 1.091^*^ | .139 | <.001 | .757 | 1.425 |
| 2 | 1 | 1.746^*^ | .180 | <.001 | 1.314 | 2.178 |
|  | 3 | 2.837^*^ | .188 | <.001 | 2.385 | 3.288 |
| 3 | 1 | -1.091^*^ | .139 | <.001 | -1.425 | -.757 |
|  | 2 | -2.837^*^ | .188 | <.001 | -3.288 | -2.385 |
| Based on estimated marginal means | | | | | | |
| *. The mean difference is significant at the .05 level. | | | | | | |
| b. Adjustment for multiple comparisons: Bonferroni. | | | | | | |

| **Multivariate Tests** | | | | | | | | |
| --- | --- | --- | --- | --- | --- | --- | --- | --- |
|  | Value | F | Hypothesis df | Error df | Sig. | Partial Eta Squared | Noncent. Parameter | Observed Power^b^ |
| Pillai's trace | .385 | 115.048^a^ | 2.000 | 367.000 | <.001 | .385 | 230.097 | 1.000 |
| Wilks' lambda | .615 | 115.048^a^ | 2.000 | 367.000 | <.001 | .385 | 230.097 | 1.000 |
| Hotelling's trace | .627 | 115.048^a^ | 2.000 | 367.000 | <.001 | .385 | 230.097 | 1.000 |
| Roy's largest root | .627 | 115.048^a^ | 2.000 | 367.000 | <.001 | .385 | 230.097 | 1.000 |
| Each F tests the multivariate effect of Stage. These tests are based on the linearly independent pairwise comparisons among the estimated marginal means. | | | | | | | | |
| a. Exact statistic | | | | | | | | |
| b. Computed using alpha = .05 | | | | | | | | |

**Positive affect**

**General Linear Model**

| **Notes** | | |
| --- | --- | --- |
| Output Created | | 29-MAR-2023 13:33:30 |
| Comments | |  |
| Input | Active Dataset | DataSet1 |
|  | Filter | <none> |
|  | Weight | <none> |
|  | Split File | <none> |
|  | N of Rows in Working Data File | 372 |
| Missing Value Handling | Definition of Missing | User-defined missing values are treated as missing. |
|  | Cases Used | Statistics are based on all cases with valid data for all variables in the model. |
| Syntax | | GLM pa_t1 pa_t2 pa_t3 BY condition /WSFACTOR=Stage 3 Simple(1) /MEASURE=Positive_affect /METHOD=SSTYPE(3) /EMMEANS=TABLES(Stage) COMPARE ADJ(BONFERRONI) /PRINT=DESCRIPTIVE ETASQ OPOWER /CRITERIA=ALPHA(.05) /WSDESIGN=Stage /DESIGN=condition. |
| Resources | Processor Time | 00:00:00.00 |
|  | Elapsed Time | 00:00:00.00 |

| **Within-Subjects Factors** | |
| --- | --- |
| Measure: Positive_affect | |
| Stage | Dependent Variable |
| 1 | pa_t1 |
| 2 | pa_t2 |
| 3 | pa_t3 |

| **Between-Subjects Factors** | | | |
| --- | --- | --- | --- |
|  | | Value Label | N |
| The experimental conditionn to which each participant was assigned | 1 | Control | 92 |
|  | 2 | plus 4 species | 90 |
|  | 3 | plus 21 species | 92 |
|  | 4 | plus 21 species and audio | 98 |

| **Descriptive Statistics** | | | | |
| --- | --- | --- | --- | --- |
|  | The experimental conditionn to which each participant was assigned | Mean | Std. Deviation | N |
| Positive Affect baseline score | Control | 14.0870 | 3.94370 | 92 |
|  | plus 4 species | 13.9889 | 4.43301 | 90 |
|  | plus 21 species | 14.0217 | 4.20094 | 92 |
|  | plus 21 species and audio | 14.5714 | 3.92980 | 98 |
|  | Total | 14.1747 | 4.11743 | 372 |
| Positive Affect score after stressor | Control | 13.9783 | 4.51123 | 92 |
|  | plus 4 species | 13.3333 | 4.88980 | 90 |
|  | plus 21 species | 13.5978 | 4.31980 | 92 |
|  | plus 21 species and audio | 13.8673 | 4.06807 | 98 |
|  | Total | 13.6989 | 4.43773 | 372 |
| Positive Affect score after video | Control | 13.4565 | 4.39883 | 92 |
|  | plus 4 species | 14.0889 | 5.17263 | 90 |
|  | plus 21 species | 13.6196 | 4.63584 | 92 |
|  | plus 21 species and audio | 13.9490 | 4.16260 | 98 |
|  | Total | 13.7796 | 4.58579 | 372 |

| **Multivariate Tests**^a^ | | | | | | | | | |
| --- | --- | --- | --- | --- | --- | --- | --- | --- | --- |
| Effect | | Value | F | Hypothesis df | Error df | Sig. | Partial Eta Squared | Noncent. Parameter | Observed Power^d^ |
| Stage | Pillai's Trace | .022 | 4.222^b^ | 2.000 | 367.000 | .015 | .022 | 8.445 | .739 |
|  | Wilks' Lambda | .978 | 4.222^b^ | 2.000 | 367.000 | .015 | .022 | 8.445 | .739 |
|  | Hotelling's Trace | .023 | 4.222^b^ | 2.000 | 367.000 | .015 | .022 | 8.445 | .739 |
|  | Roy's Largest Root | .023 | 4.222^b^ | 2.000 | 367.000 | .015 | .022 | 8.445 | .739 |
| Stage * condition | Pillai's Trace | .019 | 1.191 | 6.000 | 736.000 | .309 | .010 | 7.146 | .474 |
|  | Wilks' Lambda | .981 | 1.191^b^ | 6.000 | 734.000 | .309 | .010 | 7.147 | .474 |
|  | Hotelling's Trace | .020 | 1.191 | 6.000 | 732.000 | .309 | .010 | 7.148 | .474 |
|  | Roy's Largest Root | .017 | 2.123^c^ | 3.000 | 368.000 | .097 | .017 | 6.370 | .540 |
| a. Design: Intercept + condition Within Subjects Design: Stage | | | | | | | | | |
| b. Exact statistic | | | | | | | | | |
| c. The statistic is an upper bound on F that yields a lower bound on the significance level. | | | | | | | | | |
| d. Computed using alpha = .05 | | | | | | | | | |

| **Mauchly's Test of Sphericity**^a^ | | | | | | | |
| --- | --- | --- | --- | --- | --- | --- | --- |
| Measure: Positive_affect | | | | | | | |
| Within Subjects Effect | Mauchly's W | Approx. Chi-Square | df | Sig. | Epsilon^b^ | | |
|  |  |  |  |  | Greenhouse-Geisser | Huynh-Feldt | Lower-bound |
| Stage | .970 | 11.305 | 2 | .004 | .971 | .984 | .500 |
| Tests the null hypothesis that the error covariance matrix of the orthonormalized transformed dependent variables is proportional to an identity matrix. | | | | | | | |
| a. Design: Intercept + condition Within Subjects Design: Stage | | | | | | | |
| b. May be used to adjust the degrees of freedom for the averaged tests of significance. Corrected tests are displayed in the Tests of Within-Subjects Effects table. | | | | | | | |

| **Tests of Within-Subjects Effects** | | | | | | | | | |
| --- | --- | --- | --- | --- | --- | --- | --- | --- | --- |
| Measure: Positive_affect | | | | | | | | | |
| Source | | Type III Sum of Squares | df | Mean Square | F | Sig. | Partial Eta Squared | Noncent. Parameter | Observed Power^a^ |
| Stage | Sphericity Assumed | 47.324 | 2 | 23.662 | 3.938 | .020 | .011 | 7.877 | .709 |
|  | Greenhouse-Geisser | 47.324 | 1.941 | 24.380 | 3.938 | .021 | .011 | 7.645 | .699 |
|  | Huynh-Feldt | 47.324 | 1.967 | 24.057 | 3.938 | .020 | .011 | 7.747 | .703 |
|  | Lower-bound | 47.324 | 1.000 | 47.324 | 3.938 | .048 | .011 | 3.938 | .508 |
| Stage * condition | Sphericity Assumed | 42.529 | 6 | 7.088 | 1.180 | .315 | .010 | 7.079 | .469 |
|  | Greenhouse-Geisser | 42.529 | 5.823 | 7.303 | 1.180 | .315 | .010 | 6.870 | .461 |
|  | Huynh-Feldt | 42.529 | 5.901 | 7.206 | 1.180 | .315 | .010 | 6.962 | .465 |
|  | Lower-bound | 42.529 | 3.000 | 14.176 | 1.180 | .317 | .010 | 3.539 | .317 |
| Error(Stage) | Sphericity Assumed | 4421.896 | 736 | 6.008 |  |  |  |  |  |
|  | Greenhouse-Geisser | 4421.896 | 714.332 | 6.190 |  |  |  |  |  |
|  | Huynh-Feldt | 4421.896 | 723.915 | 6.108 |  |  |  |  |  |
|  | Lower-bound | 4421.896 | 368.000 | 12.016 |  |  |  |  |  |
| a. Computed using alpha = .05 | | | | | | | | | |

| **Tests of Within-Subjects Contrasts** | | | | | | | | | |
| --- | --- | --- | --- | --- | --- | --- | --- | --- | --- |
| Measure: Positive_affect | | | | | | | | | |
| Source | Stage | Type III Sum of Squares | df | Mean Square | F | Sig. | Partial Eta Squared | Noncent. Parameter | Observed Power^a^ |
| Stage | Level 2 vs. Level 1 | 83.165 | 1 | 83.165 | 8.071 | .005 | .021 | 8.071 | .809 |
|  | Level 3 vs. Level 1 | 56.166 | 1 | 56.166 | 4.036 | .045 | .011 | 4.036 | .518 |
| Stage * condition | Level 2 vs. Level 1 | 20.661 | 3 | 6.887 | .668 | .572 | .005 | 2.005 | .191 |
|  | Level 3 vs. Level 1 | 32.226 | 3 | 10.742 | .772 | .510 | .006 | 2.316 | .216 |
| Error(Stage) | Level 2 vs. Level 1 | 3792.121 | 368 | 10.305 |  |  |  |  |  |
|  | Level 3 vs. Level 1 | 5120.685 | 368 | 13.915 |  |  |  |  |  |
| a. Computed using alpha = .05 | | | | | | | | | |

| **Tests of Between-Subjects Effects** | | | | | | | | |
| --- | --- | --- | --- | --- | --- | --- | --- | --- |
| Measure: Positive_affect | | | | | | | | |
| Transformed Variable: Average | | | | | | | | |
| Source | Type III Sum of Squares | df | Mean Square | F | Sig. | Partial Eta Squared | Noncent. Parameter | Observed Power^a^ |
| Intercept | 71594.683 | 1 | 71594.683 | 4674.672 | <.001 | .927 | 4674.672 | 1.000 |
| condition | 8.391 | 3 | 2.797 | .183 | .908 | .001 | .548 | .084 |
| Error | 5636.083 | 368 | 15.315 |  |  |  |  |  |
| a. Computed using alpha = .05 | | | | | | | | |

**Estimated Marginal Means**

**Stage**

| **Estimates** | | | | |
| --- | --- | --- | --- | --- |
| Measure: Positive_affect | | | | |
| Stage | Mean | Std. Error | 95% Confidence Interval | |
|  |  |  | Lower Bound | Upper Bound |
| 1 | 14.167 | .214 | 13.746 | 14.588 |
| 2 | 13.694 | .231 | 13.240 | 14.148 |
| 3 | 13.778 | .238 | 13.310 | 14.247 |

| **Pairwise Comparisons** | | | | | | |
| --- | --- | --- | --- | --- | --- | --- |
| Measure: Positive_affect | | | | | | |
| (I) Stage | (J) Stage | Mean Difference (I-J) | Std. Error | Sig.^b^ | 95% Confidence Interval for Difference^b^ | |
|  |  |  |  |  | Lower Bound | Upper Bound |
| 1 | 2 | .473^*^ | .167 | .014 | .073 | .874 |
|  | 3 | .389 | .194 | .136 | -.077 | .854 |
| 2 | 1 | -.473^*^ | .167 | .014 | -.874 | -.073 |
|  | 3 | -.084 | .178 | 1.000 | -.513 | .345 |
| 3 | 1 | -.389 | .194 | .136 | -.854 | .077 |
|  | 2 | .084 | .178 | 1.000 | -.345 | .513 |
| Based on estimated marginal means | | | | | | |
| *. The mean difference is significant at the .05 level. | | | | | | |
| b. Adjustment for multiple comparisons: Bonferroni. | | | | | | |

| **Multivariate Tests** | | | | | | | | |
| --- | --- | --- | --- | --- | --- | --- | --- | --- |
|  | Value | F | Hypothesis df | Error df | Sig. | Partial Eta Squared | Noncent. Parameter | Observed Power^b^ |
| Pillai's trace | .022 | 4.222^a^ | 2.000 | 367.000 | .015 | .022 | 8.445 | .739 |
| Wilks' lambda | .978 | 4.222^a^ | 2.000 | 367.000 | .015 | .022 | 8.445 | .739 |
| Hotelling's trace | .023 | 4.222^a^ | 2.000 | 367.000 | .015 | .022 | 8.445 | .739 |
| Roy's largest root | .023 | 4.222^a^ | 2.000 | 367.000 | .015 | .022 | 8.445 | .739 |
| Each F tests the multivariate effect of Stage. These tests are based on the linearly independent pairwise comparisons among the estimated marginal means. | | | | | | | | |
| a. Exact statistic | | | | | | | | |
| b. Computed using alpha = .05 | | | | | | | | |
