# Supplementary material for: Does increasing biodiversity in an urban woodland setting promote positive emotional responses in humans? A stress recovery experiment using 360-degree videos of an urban woodland
Source: PLoS One. 2024 Feb 7;19(2):e0297179. doi: 10.1371/journal.pone.0297179 (PMC10849218; doi:10.1371/journal.pone.0297179)
Supplement: S1 Dataset — (ZIP) [file pone.0297179.s002.zip › Dataset and statistics output/3 - Perceived level of biodiversity.docx]

Affective responses and Perceived level of biodiversity

**Negative Affect**

**General Linear Model**

| **Notes** | | |
| --- | --- | --- |
| Output Created | | 29-MAR-2023 13:49:09 |
| Comments | |  |
| Input | Active Dataset | DataSet1 |
|  | Filter | <none> |
|  | Weight | <none> |
|  | Split File | <none> |
|  | N of Rows in Working Data File | 372 |
| Missing Value Handling | Definition of Missing | User-defined missing values are treated as missing. |
|  | Cases Used | Statistics are based on all cases with valid data for all variables in the model. |
| Syntax | | GLM na_t1 na_t2 na_t3 BY perc_b_2 /WSFACTOR=Stage 3 Simple(1) /MEASURE=Negative_affect /METHOD=SSTYPE(3) /POSTHOC=perc_b_2(BONFERRONI) /EMMEANS=TABLES(perc_b_2*Stage) COMPARE(perc_b_2) ADJ(BONFERRONI) /EMMEANS=TABLES(perc_b_2*Stage) COMPARE(Stage) ADJ(BONFERRONI) /PRINT=DESCRIPTIVE ETASQ OPOWER /CRITERIA=ALPHA(.05) /WSDESIGN=Stage /DESIGN=perc_b_2. |
| Resources | Processor Time | 00:00:00.00 |
|  | Elapsed Time | 00:00:00.00 |

| **Within-Subjects Factors** | |
| --- | --- |
| Measure: Negative_affect | |
| Stage | Dependent Variable |
| 1 | na_t1 |
| 2 | na_t2 |
| 3 | na_t3 |

| **Between-Subjects Factors** | | | |
| --- | --- | --- | --- |
|  | | Value Label | N |
| Perceptions of biodiversity recoded into 3 categories grouping the categories "Very bad", "bad", "Neither good nor bad" together | 3 | Bad or Neutral | 88 |
|  | 4 | Good | 197 |
|  | 5 | Very good | 87 |

| **Descriptive Statistics** | | | | |
| --- | --- | --- | --- | --- |
|  | Perceptions of biodiversity recoded into 3 categories grouping the categories "Very bad", "bad", "Neither good nor bad" together | Mean | Std. Deviation | N |
| Negative Affect baseline score | Bad or Neutral | 8.2955 | 3.86900 | 88 |
|  | Good | 7.6294 | 3.30426 | 197 |
|  | Very good | 7.4368 | 3.62710 | 87 |
|  | Total | 7.7419 | 3.52550 | 372 |
| Negative Affect score after stressor | Bad or Neutral | 10.0227 | 4.80416 | 88 |
|  | Good | 9.4416 | 3.89831 | 197 |
|  | Very good | 9.0690 | 4.25849 | 87 |
|  | Total | 9.4919 | 4.21363 | 372 |
| Negative Affect score after video | Bad or Neutral | 7.7727 | 4.03071 | 88 |
|  | Good | 6.3655 | 2.48021 | 197 |
|  | Very good | 6.1954 | 3.02255 | 87 |
|  | Total | 6.6586 | 3.09318 | 372 |

| **Multivariate Tests**^a^ | | | | | | | | | |
| --- | --- | --- | --- | --- | --- | --- | --- | --- | --- |
| Effect | | Value | F | Hypothesis df | Error df | Sig. | Partial Eta Squared | Noncent. Parameter | Observed Power^d^ |
| Stage | Pillai's Trace | .334 | 92.481^b^ | 2.000 | 368.000 | <.001 | .334 | 184.962 | 1.000 |
|  | Wilks' Lambda | .666 | 92.481^b^ | 2.000 | 368.000 | <.001 | .334 | 184.962 | 1.000 |
|  | Hotelling's Trace | .503 | 92.481^b^ | 2.000 | 368.000 | <.001 | .334 | 184.962 | 1.000 |
|  | Roy's Largest Root | .503 | 92.481^b^ | 2.000 | 368.000 | <.001 | .334 | 184.962 | 1.000 |
| Stage * perc_b_2 | Pillai's Trace | .016 | 1.485 | 4.000 | 738.000 | .205 | .008 | 5.940 | .463 |
|  | Wilks' Lambda | .984 | 1.486^b^ | 4.000 | 736.000 | .204 | .008 | 5.945 | .463 |
|  | Hotelling's Trace | .016 | 1.488 | 4.000 | 734.000 | .204 | .008 | 5.951 | .464 |
|  | Roy's Largest Root | .016 | 2.913^c^ | 2.000 | 369.000 | .056 | .016 | 5.826 | .567 |
| a. Design: Intercept + perc_b_2 Within Subjects Design: Stage | | | | | | | | | |
| b. Exact statistic | | | | | | | | | |
| c. The statistic is an upper bound on F that yields a lower bound on the significance level. | | | | | | | | | |
| d. Computed using alpha = .05 | | | | | | | | | |

| **Mauchly's Test of Sphericity**^a^ | | | | | | | |
| --- | --- | --- | --- | --- | --- | --- | --- |
| Measure: Negative_affect | | | | | | | |
| Within Subjects Effect | Mauchly's W | Approx. Chi-Square | df | Sig. | Epsilon^b^ | | |
|  |  |  |  |  | Greenhouse-Geisser | Huynh-Feldt | Lower-bound |
| Stage | .887 | 44.106 | 2 | <.001 | .899 | .908 | .500 |
| Tests the null hypothesis that the error covariance matrix of the orthonormalized transformed dependent variables is proportional to an identity matrix. | | | | | | | |
| a. Design: Intercept + perc_b_2 Within Subjects Design: Stage | | | | | | | |
| b. May be used to adjust the degrees of freedom for the averaged tests of significance. Corrected tests are displayed in the Tests of Within-Subjects Effects table. | | | | | | | |

| **Tests of Within-Subjects Effects** | | | | | | | | | |
| --- | --- | --- | --- | --- | --- | --- | --- | --- | --- |
| Measure: Negative_affect | | | | | | | | | |
| Source | | Type III Sum of Squares | df | Mean Square | F | Sig. | Partial Eta Squared | Noncent. Parameter | Observed Power^a^ |
| Stage | Sphericity Assumed | 1230.879 | 2 | 615.439 | 114.678 | <.001 | .237 | 229.355 | 1.000 |
|  | Greenhouse-Geisser | 1230.879 | 1.797 | 684.953 | 114.678 | <.001 | .237 | 206.079 | 1.000 |
|  | Huynh-Feldt | 1230.879 | 1.815 | 678.149 | 114.678 | <.001 | .237 | 208.146 | 1.000 |
|  | Lower-bound | 1230.879 | 1.000 | 1230.879 | 114.678 | <.001 | .237 | 114.678 | 1.000 |
| Stage * perc_b_2 | Sphericity Assumed | 26.657 | 4 | 6.664 | 1.242 | .292 | .007 | 4.967 | .391 |
|  | Greenhouse-Geisser | 26.657 | 3.594 | 7.417 | 1.242 | .293 | .007 | 4.463 | .368 |
|  | Huynh-Feldt | 26.657 | 3.630 | 7.343 | 1.242 | .293 | .007 | 4.508 | .370 |
|  | Lower-bound | 26.657 | 2.000 | 13.329 | 1.242 | .290 | .007 | 2.484 | .270 |
| Error(Stage) | Sphericity Assumed | 3960.621 | 738 | 5.367 |  |  |  |  |  |
|  | Greenhouse-Geisser | 3960.621 | 663.103 | 5.973 |  |  |  |  |  |
|  | Huynh-Feldt | 3960.621 | 669.756 | 5.914 |  |  |  |  |  |
|  | Lower-bound | 3960.621 | 369.000 | 10.733 |  |  |  |  |  |
| a. Computed using alpha = .05 | | | | | | | | | |

| **Tests of Within-Subjects Contrasts** | | | | | | | | | |
| --- | --- | --- | --- | --- | --- | --- | --- | --- | --- |
| Measure: Negative_affect | | | | | | | | | |
| Source | Stage | Type III Sum of Squares | df | Mean Square | F | Sig. | Partial Eta Squared | Noncent. Parameter | Observed Power^a^ |
| Stage | Level 2 vs. Level 1 | 957.465 | 1 | 957.465 | 80.047 | <.001 | .178 | 80.047 | 1.000 |
|  | Level 3 vs. Level 1 | 328.244 | 1 | 328.244 | 45.704 | <.001 | .110 | 45.704 | 1.000 |
| Stage * perc_b_2 | Level 2 vs. Level 1 | 2.015 | 2 | 1.007 | .084 | .919 | .000 | .168 | .063 |
|  | Level 3 vs. Level 1 | 36.257 | 2 | 18.128 | 2.524 | .082 | .013 | 5.048 | .504 |
| Error(Stage) | Level 2 vs. Level 1 | 4413.735 | 369 | 11.961 |  |  |  |  |  |
|  | Level 3 vs. Level 1 | 2650.160 | 369 | 7.182 |  |  |  |  |  |
| a. Computed using alpha = .05 | | | | | | | | | |

| **Tests of Between-Subjects Effects** | | | | | | | | |
| --- | --- | --- | --- | --- | --- | --- | --- | --- |
| Measure: Negative_affect | | | | | | | | |
| Transformed Variable: Average | | | | | | | | |
| Source | Type III Sum of Squares | df | Mean Square | F | Sig. | Partial Eta Squared | Noncent. Parameter | Observed Power^a^ |
| Intercept | 20751.159 | 1 | 20751.159 | 2174.514 | <.001 | .855 | 2174.514 | 1.000 |
| perc_b_2 | 65.527 | 2 | 32.763 | 3.433 | .033 | .018 | 6.867 | .643 |
| Error | 3521.329 | 369 | 9.543 |  |  |  |  |  |
| a. Computed using alpha = .05 | | | | | | | | |

**Estimated Marginal Means**

**1. Perceptions of biodiversity recoded * Stage**

| **Estimates** | | | | | |
| --- | --- | --- | --- | --- | --- |
| Measure: Negative_affect | | | | | |
| Perceptions of biodiversity recoded into 3 categories grouping the categories "Very bad", "bad", "Neither good nor bad" together | Stage | Mean | Std. Error | 95% Confidence Interval | |
|  |  |  |  | Lower Bound | Upper Bound |
| Bad or Neutral | 1 | 8.295 | .375 | 7.557 | 9.033 |
|  | 2 | 10.023 | .449 | 9.140 | 10.906 |
|  | 3 | 7.773 | .324 | 7.136 | 8.409 |
| Good | 1 | 7.629 | .251 | 7.136 | 8.123 |
|  | 2 | 9.442 | .300 | 8.852 | 10.032 |
|  | 3 | 6.365 | .216 | 5.940 | 6.791 |
| Very good | 1 | 7.437 | .377 | 6.695 | 8.179 |
|  | 2 | 9.069 | .452 | 8.181 | 9.957 |
|  | 3 | 6.195 | .326 | 5.555 | 6.836 |

| **Pairwise Comparisons** | | | | | | | |
| --- | --- | --- | --- | --- | --- | --- | --- |
| Measure: Negative_affect | | | | | | | |
| Stage | (I) Perceptions of biodiversity recoded | (J) Perceptions of biodiversity recoded | Mean Difference (I-J) | Std. Error | Sig.^b^ | 95% Confidence Interval for Difference^b^ | |
|  |  |  |  |  |  | Lower Bound | Upper Bound |
| 1 | Bad or Neutral | Good | .666 | .451 | .423 | -.420 | 1.752 |
|  |  | Very good | .859 | .532 | .323 | -.421 | 2.139 |
|  | Good | Bad or Neutral | -.666 | .451 | .423 | -1.752 | .420 |
|  |  | Very good | .193 | .453 | 1.000 | -.897 | 1.283 |
|  | Very good | Bad or Neutral | -.859 | .532 | .323 | -2.139 | .421 |
|  |  | Good | -.193 | .453 | 1.000 | -1.283 | .897 |
| 2 | Bad or Neutral | Good | .581 | .540 | .848 | -.718 | 1.880 |
|  |  | Very good | .954 | .637 | .405 | -.578 | 2.485 |
|  | Good | Bad or Neutral | -.581 | .540 | .848 | -1.880 | .718 |
|  |  | Very good | .373 | .542 | 1.000 | -.931 | 1.677 |
|  | Very good | Bad or Neutral | -.954 | .637 | .405 | -2.485 | .578 |
|  |  | Good | -.373 | .542 | 1.000 | -1.677 | .931 |
| 3 | Bad or Neutral | Good | 1.407^*^ | .389 | .001 | .471 | 2.344 |
|  |  | Very good | 1.577^*^ | .459 | .002 | .473 | 2.682 |
|  | Good | Bad or Neutral | -1.407^*^ | .389 | .001 | -2.344 | -.471 |
|  |  | Very good | .170 | .391 | 1.000 | -.770 | 1.110 |
|  | Very good | Bad or Neutral | -1.577^*^ | .459 | .002 | -2.682 | -.473 |
|  |  | Good | -.170 | .391 | 1.000 | -1.110 | .770 |
| Based on estimated marginal means | | | | | | | |
| *. The mean difference is significant at the .05 level. | | | | | | | |
| b. Adjustment for multiple comparisons: Bonferroni. | | | | | | | |

| **Univariate Tests** | | | | | | | | | |
| --- | --- | --- | --- | --- | --- | --- | --- | --- | --- |
| Measure: Negative_affect | | | | | | | | | |
| Stage | | Sum of Squares | df | Mean Square | F | Sig. | Partial Eta Squared | Noncent. Parameter | Observed Power^a^ |
| 1 | Contrast | 37.556 | 2 | 18.778 | 1.515 | .221 | .008 | 3.030 | .322 |
|  | Error | 4573.670 | 369 | 12.395 |  |  |  |  |  |
| 2 | Contrast | 40.856 | 2 | 20.428 | 1.152 | .317 | .006 | 2.303 | .253 |
|  | Error | 6546.119 | 369 | 17.740 |  |  |  |  |  |
| 3 | Contrast | 144.824 | 2 | 72.412 | 7.848 | <.001 | .041 | 15.695 | .952 |
|  | Error | 3404.818 | 369 | 9.227 |  |  |  |  |  |
| Each F tests the simple effects of Perceptions of biodiversity recoded within each level combination of the other effects shown. These tests are based on the linearly independent pairwise comparisons among the estimated marginal means. | | | | | | | | | |
| a. Computed using alpha = .05 | | | | | | | | | |

**2. Perceptions of biodiversity recoded * Stage**

| **Estimates** | | | | | |
| --- | --- | --- | --- | --- | --- |
| Measure: Negative_affect | | | | | |
| Perceptions of biodiversity recoded into 3 categories grouping the categories "Very bad", "bad", "Neither good nor bad" together | Stage | Mean | Std. Error | 95% Confidence Interval | |
|  |  |  |  | Lower Bound | Upper Bound |
| Bad or Neutral | 1 | 8.295 | .375 | 7.557 | 9.033 |
|  | 2 | 10.023 | .449 | 9.140 | 10.906 |
|  | 3 | 7.773 | .324 | 7.136 | 8.409 |
| Good | 1 | 7.629 | .251 | 7.136 | 8.123 |
|  | 2 | 9.442 | .300 | 8.852 | 10.032 |
|  | 3 | 6.365 | .216 | 5.940 | 6.791 |
| Very good | 1 | 7.437 | .377 | 6.695 | 8.179 |
|  | 2 | 9.069 | .452 | 8.181 | 9.957 |
|  | 3 | 6.195 | .326 | 5.555 | 6.836 |

| **Pairwise Comparisons** | | | | | | | |
| --- | --- | --- | --- | --- | --- | --- | --- |
| Measure: Negative_affect | | | | | | | |
| Perceptions of biodiversity recoded into 3 categories grouping the categories "Very bad", "bad", "Neither good nor bad" together | (I) Stage | (J) Stage | Mean Difference (I-J) | Std. Error | Sig.^b^ | 95% Confidence Interval for Difference^b^ | |
|  |  |  |  |  |  | Lower Bound | Upper Bound |
| Bad or Neutral | 1 | 2 | -1.727^*^ | .369 | <.001 | -2.614 | -.841 |
|  |  | 3 | .523 | .286 | .204 | -.164 | 1.210 |
|  | 2 | 1 | 1.727^*^ | .369 | <.001 | .841 | 2.614 |
|  |  | 3 | 2.250^*^ | .385 | <.001 | 1.324 | 3.176 |
|  | 3 | 1 | -.523 | .286 | .204 | -1.210 | .164 |
|  |  | 2 | -2.250^*^ | .385 | <.001 | -3.176 | -1.324 |
| Good | 1 | 2 | -1.812^*^ | .246 | <.001 | -2.405 | -1.220 |
|  |  | 3 | 1.264^*^ | .191 | <.001 | .805 | 1.723 |
|  | 2 | 1 | 1.812^*^ | .246 | <.001 | 1.220 | 2.405 |
|  |  | 3 | 3.076^*^ | .257 | <.001 | 2.457 | 3.695 |
|  | 3 | 1 | -1.264^*^ | .191 | <.001 | -1.723 | -.805 |
|  |  | 2 | -3.076^*^ | .257 | <.001 | -3.695 | -2.457 |
| Very good | 1 | 2 | -1.632^*^ | .371 | <.001 | -2.524 | -.740 |
|  |  | 3 | 1.241^*^ | .287 | <.001 | .550 | 1.932 |
|  | 2 | 1 | 1.632^*^ | .371 | <.001 | .740 | 2.524 |
|  |  | 3 | 2.874^*^ | .387 | <.001 | 1.942 | 3.805 |
|  | 3 | 1 | -1.241^*^ | .287 | <.001 | -1.932 | -.550 |
|  |  | 2 | -2.874^*^ | .387 | <.001 | -3.805 | -1.942 |
| Based on estimated marginal means | | | | | | | |
| *. The mean difference is significant at the .05 level. | | | | | | | |
| b. Adjustment for multiple comparisons: Bonferroni. | | | | | | | |

| **Multivariate Tests** | | | | | | | | | |
| --- | --- | --- | --- | --- | --- | --- | --- | --- | --- |
| Perceptions of biodiversity recoded into 3 categories grouping the categories "Very bad", "bad", "Neither good nor bad" together | | Value | F | Hypothesis df | Error df | Sig. | Partial Eta Squared | Noncent. Parameter | Observed Power^b^ |
| Bad or Neutral | Pillai's trace | .086 | 17.285^a^ | 2.000 | 368.000 | <.001 | .086 | 34.570 | 1.000 |
|  | Wilks' lambda | .914 | 17.285^a^ | 2.000 | 368.000 | <.001 | .086 | 34.570 | 1.000 |
|  | Hotelling's trace | .094 | 17.285^a^ | 2.000 | 368.000 | <.001 | .086 | 34.570 | 1.000 |
|  | Roy's largest root | .094 | 17.285^a^ | 2.000 | 368.000 | <.001 | .086 | 34.570 | 1.000 |
| Good | Pillai's trace | .283 | 72.589^a^ | 2.000 | 368.000 | <.001 | .283 | 145.178 | 1.000 |
|  | Wilks' lambda | .717 | 72.589^a^ | 2.000 | 368.000 | <.001 | .283 | 145.178 | 1.000 |
|  | Hotelling's trace | .395 | 72.589^a^ | 2.000 | 368.000 | <.001 | .283 | 145.178 | 1.000 |
|  | Roy's largest root | .395 | 72.589^a^ | 2.000 | 368.000 | <.001 | .283 | 145.178 | 1.000 |
| Very good | Pillai's trace | .133 | 28.243^a^ | 2.000 | 368.000 | <.001 | .133 | 56.486 | 1.000 |
|  | Wilks' lambda | .867 | 28.243^a^ | 2.000 | 368.000 | <.001 | .133 | 56.486 | 1.000 |
|  | Hotelling's trace | .153 | 28.243^a^ | 2.000 | 368.000 | <.001 | .133 | 56.486 | 1.000 |
|  | Roy's largest root | .153 | 28.243^a^ | 2.000 | 368.000 | <.001 | .133 | 56.486 | 1.000 |
| Each F tests the multivariate simple effects of Stage within each level combination of the other effects shown. These tests are based on the linearly independent pairwise comparisons among the estimated marginal means. | | | | | | | | | |
| a. Exact statistic | | | | | | | | | |
| b. Computed using alpha = .05 | | | | | | | | | |

**Post Hoc Tests**

**Perceptions of biodiversity recoded into 3 categories grouping the categories "Very bad", "bad", "Neither good nor bad" together**

| **Multiple Comparisons** | | | | | | |
| --- | --- | --- | --- | --- | --- | --- |
| Measure: Negative_affect | | | | | | |
| Bonferroni | | | | | | |
| (I) Perceptions of biodiversity recoded into 3 categories grouping the categories "Very bad", "bad", "Neither good nor bad" together | (J) Perceptions of biodiversity recoded into 3 categories grouping the categories "Very bad", "bad", "Neither good nor bad" together | Mean Difference (I-J) | Std. Error | Sig. | 95% Confidence Interval | |
|  |  |  |  |  | Lower Bound | Upper Bound |
| Bad or Neutral | Good | .8848 | .39608 | .078 | -.0678 | 1.8373 |
|  | Very good | 1.1299^*^ | .46704 | .048 | .0067 | 2.2531 |
| Good | Bad or Neutral | -.8848 | .39608 | .078 | -1.8373 | .0678 |
|  | Very good | .2451 | .39766 | 1.000 | -.7112 | 1.2015 |
| Very good | Bad or Neutral | -1.1299^*^ | .46704 | .048 | -2.2531 | -.0067 |
|  | Good | -.2451 | .39766 | 1.000 | -1.2015 | .7112 |
| Based on observed means. The error term is Mean Square(Error) = 9.543. | | | | | | |
| *. The mean difference is significant at the .05 level. | | | | | | |

**Positive Affect**

**General Linear Model**

| **Notes** | | |
| --- | --- | --- |
| Output Created | | 29-MAR-2023 13:50:13 |
| Comments | |  |
| Input | Active Dataset | DataSet1 |
|  | Filter | <none> |
|  | Weight | <none> |
|  | Split File | <none> |
|  | N of Rows in Working Data File | 372 |
| Missing Value Handling | Definition of Missing | User-defined missing values are treated as missing. |
|  | Cases Used | Statistics are based on all cases with valid data for all variables in the model. |
| Syntax | | GLM pa_t1 pa_t2 pa_t3 BY perc_b_2 /WSFACTOR=Stage 3 Simple(1) /MEASURE=Positive_affect /METHOD=SSTYPE(3) /POSTHOC=perc_b_2(BONFERRONI) /EMMEANS=TABLES(perc_b_2*Stage) COMPARE(perc_b_2) ADJ(BONFERRONI) /EMMEANS=TABLES(perc_b_2*Stage) COMPARE(Stage) ADJ(BONFERRONI) /PRINT=DESCRIPTIVE ETASQ OPOWER /CRITERIA=ALPHA(.05) /WSDESIGN=Stage /DESIGN=perc_b_2. |
| Resources | Processor Time | 00:00:00.00 |
|  | Elapsed Time | 00:00:00.00 |

| **Within-Subjects Factors** | |
| --- | --- |
| Measure: Positive_affect | |
| Stage | Dependent Variable |
| 1 | pa_t1 |
| 2 | pa_t2 |
| 3 | pa_t3 |

| **Between-Subjects Factors** | | | |
| --- | --- | --- | --- |
|  | | Value Label | N |
| Perceptions of biodiversity recoded into 3 categories grouping the categories "Very bad", "bad", "Neither good nor bad" together | 3 | Bad or Neutral | 88 |
|  | 4 | Good | 197 |
|  | 5 | Very good | 87 |

| **Descriptive Statistics** | | | | |
| --- | --- | --- | --- | --- |
|  | Perceptions of biodiversity recoded into 3 categories grouping the categories "Very bad", "bad", "Neither good nor bad" together | Mean | Std. Deviation | N |
| Positive Affect baseline score | Bad or Neutral | 14.1591 | 3.97662 | 88 |
|  | Good | 13.8020 | 3.94042 | 197 |
|  | Very good | 15.0345 | 4.54806 | 87 |
|  | Total | 14.1747 | 4.11743 | 372 |
| Positive Affect score after stressor | Bad or Neutral | 13.7273 | 4.52763 | 88 |
|  | Good | 13.1574 | 4.14663 | 197 |
|  | Very good | 14.8966 | 4.78621 | 87 |
|  | Total | 13.6989 | 4.43773 | 372 |
| Positive Affect score after video | Bad or Neutral | 12.5114 | 4.65350 | 88 |
|  | Good | 13.4772 | 4.26970 | 197 |
|  | Very good | 15.7471 | 4.63610 | 87 |
|  | Total | 13.7796 | 4.58579 | 372 |

| **Multivariate Tests**^a^ | | | | | | | | | |
| --- | --- | --- | --- | --- | --- | --- | --- | --- | --- |
| Effect | | Value | F | Hypothesis df | Error df | Sig. | Partial Eta Squared | Noncent. Parameter | Observed Power^d^ |
| Stage | Pillai's Trace | .017 | 3.099^b^ | 2.000 | 368.000 | .046 | .017 | 6.199 | .595 |
|  | Wilks' Lambda | .983 | 3.099^b^ | 2.000 | 368.000 | .046 | .017 | 6.199 | .595 |
|  | Hotelling's Trace | .017 | 3.099^b^ | 2.000 | 368.000 | .046 | .017 | 6.199 | .595 |
|  | Roy's Largest Root | .017 | 3.099^b^ | 2.000 | 368.000 | .046 | .017 | 6.199 | .595 |
| Stage * perc_b_2 | Pillai's Trace | .062 | 5.931 | 4.000 | 738.000 | <.001 | .031 | 23.722 | .985 |
|  | Wilks' Lambda | .938 | 5.990^b^ | 4.000 | 736.000 | <.001 | .032 | 23.961 | .986 |
|  | Hotelling's Trace | .066 | 6.049 | 4.000 | 734.000 | <.001 | .032 | 24.198 | .986 |
|  | Roy's Largest Root | .062 | 11.462^c^ | 2.000 | 369.000 | <.001 | .058 | 22.923 | .993 |
| a. Design: Intercept + perc_b_2 Within Subjects Design: Stage | | | | | | | | | |
| b. Exact statistic | | | | | | | | | |
| c. The statistic is an upper bound on F that yields a lower bound on the significance level. | | | | | | | | | |
| d. Computed using alpha = .05 | | | | | | | | | |

| **Mauchly's Test of Sphericity**^a^ | | | | | | | |
| --- | --- | --- | --- | --- | --- | --- | --- |
| Measure: Positive_affect | | | | | | | |
| Within Subjects Effect | Mauchly's W | Approx. Chi-Square | df | Sig. | Epsilon^b^ | | |
|  |  |  |  |  | Greenhouse-Geisser | Huynh-Feldt | Lower-bound |
| Stage | .977 | 8.405 | 2 | .015 | .978 | .988 | .500 |
| Tests the null hypothesis that the error covariance matrix of the orthonormalized transformed dependent variables is proportional to an identity matrix. | | | | | | | |
| a. Design: Intercept + perc_b_2 Within Subjects Design: Stage | | | | | | | |
| b. May be used to adjust the degrees of freedom for the averaged tests of significance. Corrected tests are displayed in the Tests of Within-Subjects Effects table. | | | | | | | |

| **Tests of Within-Subjects Effects** | | | | | | | | | |
| --- | --- | --- | --- | --- | --- | --- | --- | --- | --- |
| Measure: Positive_affect | | | | | | | | | |
| Source | | Type III Sum of Squares | df | Mean Square | F | Sig. | Partial Eta Squared | Noncent. Parameter | Observed Power^a^ |
| Stage | Sphericity Assumed | 36.567 | 2 | 18.283 | 3.133 | .044 | .008 | 6.266 | .602 |
|  | Greenhouse-Geisser | 36.567 | 1.956 | 18.696 | 3.133 | .045 | .008 | 6.127 | .595 |
|  | Huynh-Feldt | 36.567 | 1.977 | 18.498 | 3.133 | .045 | .008 | 6.193 | .599 |
|  | Lower-bound | 36.567 | 1.000 | 36.567 | 3.133 | .078 | .008 | 3.133 | .423 |
| Stage * perc_b_2 | Sphericity Assumed | 157.433 | 4 | 39.358 | 6.744 | <.001 | .035 | 26.976 | .993 |
|  | Greenhouse-Geisser | 157.433 | 3.912 | 40.247 | 6.744 | <.001 | .035 | 26.380 | .992 |
|  | Huynh-Feldt | 157.433 | 3.954 | 39.820 | 6.744 | <.001 | .035 | 26.663 | .993 |
|  | Lower-bound | 157.433 | 2.000 | 78.717 | 6.744 | .001 | .035 | 13.488 | .916 |
| Error(Stage) | Sphericity Assumed | 4306.991 | 738 | 5.836 |  |  |  |  |  |
|  | Greenhouse-Geisser | 4306.991 | 721.704 | 5.968 |  |  |  |  |  |
|  | Huynh-Feldt | 4306.991 | 729.438 | 5.905 |  |  |  |  |  |
|  | Lower-bound | 4306.991 | 369.000 | 11.672 |  |  |  |  |  |
| a. Computed using alpha = .05 | | | | | | | | | |

| **Tests of Within-Subjects Contrasts** | | | | | | | | | |
| --- | --- | --- | --- | --- | --- | --- | --- | --- | --- |
| Measure: Positive_affect | | | | | | | | | |
| Source | Stage | Type III Sum of Squares | df | Mean Square | F | Sig. | Partial Eta Squared | Noncent. Parameter | Observed Power^a^ |
| Stage | Level 2 vs. Level 1 | 52.796 | 1 | 52.796 | 5.131 | .024 | .014 | 5.131 | .618 |
|  | Level 3 vs. Level 1 | 56.830 | 1 | 56.830 | 4.273 | .039 | .011 | 4.273 | .541 |
| Stage * perc_b_2 | Level 2 vs. Level 1 | 15.720 | 2 | 7.860 | .764 | .467 | .004 | 1.528 | .180 |
|  | Level 3 vs. Level 1 | 245.808 | 2 | 122.904 | 9.242 | <.001 | .048 | 18.484 | .977 |
| Error(Stage) | Level 2 vs. Level 1 | 3797.063 | 369 | 10.290 |  |  |  |  |  |
|  | Level 3 vs. Level 1 | 4907.104 | 369 | 13.298 |  |  |  |  |  |
| a. Computed using alpha = .05 | | | | | | | | | |

| **Tests of Between-Subjects Effects** | | | | | | | | |
| --- | --- | --- | --- | --- | --- | --- | --- | --- |
| Measure: Positive_affect | | | | | | | | |
| Transformed Variable: Average | | | | | | | | |
| Source | Type III Sum of Squares | df | Mean Square | F | Sig. | Partial Eta Squared | Noncent. Parameter | Observed Power^a^ |
| Intercept | 63663.471 | 1 | 63663.471 | 4318.303 | <.001 | .921 | 4318.303 | 1.000 |
| perc_b_2 | 204.416 | 2 | 102.208 | 6.933 | .001 | .036 | 13.866 | .923 |
| Error | 5440.058 | 369 | 14.743 |  |  |  |  |  |
| a. Computed using alpha = .05 | | | | | | | | |

**Estimated Marginal Means**

**1. Perceptions of biodiversity recoded * Stage**

| **Estimates** | | | | | |
| --- | --- | --- | --- | --- | --- |
| Measure: Positive_affect | | | | | |
| Perceptions of biodiversity recoded into 3 categories grouping the categories "Very bad", "bad", "Neither good nor bad" together | Stage | Mean | Std. Error | 95% Confidence Interval | |
|  |  |  |  | Lower Bound | Upper Bound |
| Bad or Neutral | 1 | 14.159 | .437 | 13.300 | 15.018 |
|  | 2 | 13.727 | .468 | 12.806 | 14.648 |
|  | 3 | 12.511 | .474 | 11.579 | 13.444 |
| Good | 1 | 13.802 | .292 | 13.228 | 14.376 |
|  | 2 | 13.157 | .313 | 12.542 | 13.773 |
|  | 3 | 13.477 | .317 | 12.854 | 14.101 |
| Very good | 1 | 15.034 | .439 | 14.170 | 15.899 |
|  | 2 | 14.897 | .471 | 13.970 | 15.823 |
|  | 3 | 15.747 | .477 | 14.809 | 16.685 |

| **Pairwise Comparisons** | | | | | | | |
| --- | --- | --- | --- | --- | --- | --- | --- |
| Measure: Positive_affect | | | | | | | |
| Stage | (I) Perceptions of biodiversity recoded | (J) Perceptions of biodiversity recoded | Mean Difference (I-J) | Std. Error | Sig.^b^ | 95% Confidence Interval for Difference^b^ | |
|  |  |  |  |  |  | Lower Bound | Upper Bound |
| 1 | Bad or Neutral | Good | .357 | .525 | 1.000 | -.907 | 1.621 |
|  |  | Very good | -.875 | .620 | .476 | -2.366 | .615 |
|  | Good | Bad or Neutral | -.357 | .525 | 1.000 | -1.621 | .907 |
|  |  | Very good | -1.232 | .528 | .060 | -2.501 | .036 |
|  | Very good | Bad or Neutral | .875 | .620 | .476 | -.615 | 2.366 |
|  |  | Good | 1.232 | .528 | .060 | -.036 | 2.501 |
| 2 | Bad or Neutral | Good | .570 | .563 | .937 | -.785 | 1.925 |
|  |  | Very good | -1.169 | .664 | .238 | -2.767 | .428 |
|  | Good | Bad or Neutral | -.570 | .563 | .937 | -1.925 | .785 |
|  |  | Very good | -1.739^*^ | .566 | .007 | -3.099 | -.379 |
|  | Very good | Bad or Neutral | 1.169 | .664 | .238 | -.428 | 2.767 |
|  |  | Good | 1.739^*^ | .566 | .007 | .379 | 3.099 |
| 3 | Bad or Neutral | Good | -.966 | .571 | .274 | -2.338 | .406 |
|  |  | Very good | -3.236^*^ | .673 | <.001 | -4.854 | -1.618 |
|  | Good | Bad or Neutral | .966 | .571 | .274 | -.406 | 2.338 |
|  |  | Very good | -2.270^*^ | .573 | <.001 | -3.647 | -.892 |
|  | Very good | Bad or Neutral | 3.236^*^ | .673 | <.001 | 1.618 | 4.854 |
|  |  | Good | 2.270^*^ | .573 | <.001 | .892 | 3.647 |
| Based on estimated marginal means | | | | | | | |
| *. The mean difference is significant at the .05 level. | | | | | | | |
| b. Adjustment for multiple comparisons: Bonferroni. | | | | | | | |

| **Univariate Tests** | | | | | | | | | |
| --- | --- | --- | --- | --- | --- | --- | --- | --- | --- |
| Measure: Positive_affect | | | | | | | | | |
| Stage | | Sum of Squares | df | Mean Square | F | Sig. | Partial Eta Squared | Noncent. Parameter | Observed Power^a^ |
| 1 | Contrast | 91.694 | 2 | 45.847 | 2.730 | .067 | .015 | 5.459 | .538 |
|  | Error | 6197.948 | 369 | 16.797 |  |  |  |  |  |
| 2 | Contrast | 182.634 | 2 | 91.317 | 4.730 | .009 | .025 | 9.460 | .789 |
|  | Error | 7123.645 | 369 | 19.305 |  |  |  |  |  |
| 3 | Contrast | 496.352 | 2 | 248.176 | 12.535 | <.001 | .064 | 25.070 | .996 |
|  | Error | 7305.573 | 369 | 19.798 |  |  |  |  |  |
| Each F tests the simple effects of Perceptions of biodiversity recoded into 3 categories grouping the categories "Very bad", "bad", "Neither good nor bad" together within each level combination of the other effects shown. These tests are based on the linearly independent pairwise comparisons among the estimated marginal means. | | | | | | | | | |
| a. Computed using alpha = .05 | | | | | | | | | |

**2. Perceptions of biodiversity recoded * Stage**

| **Estimates** | | | | | |
| --- | --- | --- | --- | --- | --- |
| Measure: Positive_affect | | | | | |
| Perceptions of biodiversity recoded into 3 categories grouping the categories "Very bad", "bad", "Neither good nor bad" together | Stage | Mean | Std. Error | 95% Confidence Interval | |
|  |  |  |  | Lower Bound | Upper Bound |
| Bad or Neutral | 1 | 14.159 | .437 | 13.300 | 15.018 |
|  | 2 | 13.727 | .468 | 12.806 | 14.648 |
|  | 3 | 12.511 | .474 | 11.579 | 13.444 |
| Good | 1 | 13.802 | .292 | 13.228 | 14.376 |
|  | 2 | 13.157 | .313 | 12.542 | 13.773 |
|  | 3 | 13.477 | .317 | 12.854 | 14.101 |
| Very good | 1 | 15.034 | .439 | 14.170 | 15.899 |
|  | 2 | 14.897 | .471 | 13.970 | 15.823 |
|  | 3 | 15.747 | .477 | 14.809 | 16.685 |

| **Pairwise Comparisons** | | | | | | | |
| --- | --- | --- | --- | --- | --- | --- | --- |
| Measure: Positive_affect | | | | | | | |
| Perceptions of biodiversity recoded into 3 categories grouping the categories "Very bad", "bad", "Neither good nor bad" together | (I) Stage | (J) Stage | Mean Difference (I-J) | Std. Error | Sig.^b^ | 95% Confidence Interval for Difference^b^ | |
|  |  |  |  |  |  | Lower Bound | Upper Bound |
| Bad or Neutral | 1 | 2 | .432 | .342 | .622 | -.391 | 1.254 |
|  |  | 3 | 1.648^*^ | .389 | <.001 | .713 | 2.583 |
|  | 2 | 1 | -.432 | .342 | .622 | -1.254 | .391 |
|  |  | 3 | 1.216^*^ | .360 | .002 | .349 | 2.083 |
|  | 3 | 1 | -1.648^*^ | .389 | <.001 | -2.583 | -.713 |
|  |  | 2 | -1.216^*^ | .360 | .002 | -2.083 | -.349 |
| Good | 1 | 2 | .645^*^ | .229 | .015 | .095 | 1.194 |
|  |  | 3 | .325 | .260 | .636 | -.300 | .950 |
|  | 2 | 1 | -.645^*^ | .229 | .015 | -1.194 | -.095 |
|  |  | 3 | -.320 | .241 | .555 | -.899 | .259 |
|  | 3 | 1 | -.325 | .260 | .636 | -.950 | .300 |
|  |  | 2 | .320 | .241 | .555 | -.259 | .899 |
| Very good | 1 | 2 | .138 | .344 | 1.000 | -.689 | .965 |
|  |  | 3 | -.713 | .391 | .207 | -1.653 | .228 |
|  | 2 | 1 | -.138 | .344 | 1.000 | -.965 | .689 |
|  |  | 3 | -.851 | .362 | .058 | -1.722 | .021 |
|  | 3 | 1 | .713 | .391 | .207 | -.228 | 1.653 |
|  |  | 2 | .851 | .362 | .058 | -.021 | 1.722 |
| Based on estimated marginal means | | | | | | | |
| *. The mean difference is significant at the .05 level. | | | | | | | |
| b. Adjustment for multiple comparisons: Bonferroni. | | | | | | | |

| **Multivariate Tests** | | | | | | | | | |
| --- | --- | --- | --- | --- | --- | --- | --- | --- | --- |
| Perceptions of biodiversity recoded into 3 categories grouping the categories "Very bad", "bad", "Neither good nor bad" together | | Value | F | Hypothesis df | Error df | Sig. | Partial Eta Squared | Noncent. Parameter | Observed Power^b^ |
| Bad or Neutral | Pillai's trace | .049 | 9.563^a^ | 2.000 | 368.000 | <.001 | .049 | 19.126 | .980 |
|  | Wilks' lambda | .951 | 9.563^a^ | 2.000 | 368.000 | <.001 | .049 | 19.126 | .980 |
|  | Hotelling's trace | .052 | 9.563^a^ | 2.000 | 368.000 | <.001 | .049 | 19.126 | .980 |
|  | Roy's largest root | .052 | 9.563^a^ | 2.000 | 368.000 | <.001 | .049 | 19.126 | .980 |
| Good | Pillai's trace | .021 | 3.999^a^ | 2.000 | 368.000 | .019 | .021 | 7.999 | .714 |
|  | Wilks' lambda | .979 | 3.999^a^ | 2.000 | 368.000 | .019 | .021 | 7.999 | .714 |
|  | Hotelling's trace | .022 | 3.999^a^ | 2.000 | 368.000 | .019 | .021 | 7.999 | .714 |
|  | Roy's largest root | .022 | 3.999^a^ | 2.000 | 368.000 | .019 | .021 | 7.999 | .714 |
| Very good | Pillai's trace | .016 | 2.899^a^ | 2.000 | 368.000 | .056 | .016 | 5.798 | .565 |
|  | Wilks' lambda | .984 | 2.899^a^ | 2.000 | 368.000 | .056 | .016 | 5.798 | .565 |
|  | Hotelling's trace | .016 | 2.899^a^ | 2.000 | 368.000 | .056 | .016 | 5.798 | .565 |
|  | Roy's largest root | .016 | 2.899^a^ | 2.000 | 368.000 | .056 | .016 | 5.798 | .565 |
| Each F tests the multivariate simple effects of Stage within each level combination of the other effects shown. These tests are based on the linearly independent pairwise comparisons among the estimated marginal means. | | | | | | | | | |
| a. Exact statistic | | | | | | | | | |
| b. Computed using alpha = .05 | | | | | | | | | |

**Post Hoc Tests**

**Perceptions of biodiversity recoded into 3 categories grouping the categories "Very bad", "bad", "Neither good nor bad" together**

| **Multiple Comparisons** | | | | | | |
| --- | --- | --- | --- | --- | --- | --- |
| Measure: Positive_affect | | | | | | |
| Bonferroni | | | | | | |
| (I) Perceptions of biodiversity recoded into 3 categories grouping the categories "Very bad", "bad", "Neither good nor bad" together | (J) Perceptions of biodiversity recoded into 3 categories grouping the categories "Very bad", "bad", "Neither good nor bad" together | Mean Difference (I-J) | Std. Error | Sig. | 95% Confidence Interval | |
|  |  |  |  |  | Lower Bound | Upper Bound |
| Bad or Neutral | Good | -.0129 | .49231 | 1.000 | -1.1969 | 1.1710 |
|  | Very good | -1.7601^*^ | .58051 | .008 | -3.1562 | -.3641 |
| Good | Bad or Neutral | .0129 | .49231 | 1.000 | -1.1710 | 1.1969 |
|  | Very good | -1.7472^*^ | .49426 | .001 | -2.9359 | -.5585 |
| Very good | Bad or Neutral | 1.7601^*^ | .58051 | .008 | .3641 | 3.1562 |
|  | Good | 1.7472^*^ | .49426 | .001 | .5585 | 2.9359 |
| Based on observed means. The error term is Mean Square(Error) = 14.743. | | | | | | |
| *. The mean difference is significant at the .05 level. | | | | | | |
