# Supplementary material for: Does increasing biodiversity in an urban woodland setting promote positive emotional responses in humans? A stress recovery experiment using 360-degree videos of an urban woodland
Source: PLoS One. 2024 Feb 7;19(2):e0297179. doi: 10.1371/journal.pone.0297179 (PMC10849218; doi:10.1371/journal.pone.0297179)
Supplement: S1 Dataset — (ZIP) [file pone.0297179.s002.zip › Dataset and statistics output/4 - Appreciating individual elements of nature.docx]

Affective responses and appreciating individual elements of nature

**Noticing Flowers**

**Negative Affect**

**General Linear Model**

| **Notes** | | |
| --- | --- | --- |
| Output Created | | 29-MAR-2023 13:58:28 |
| Comments | |  |
| Input | Active Dataset | DataSet1 |
|  | Filter | <none> |
|  | Weight | <none> |
|  | Split File | <none> |
|  | N of Rows in Working Data File | 372 |
| Missing Value Handling | Definition of Missing | User-defined missing values are treated as missing. |
|  | Cases Used | Statistics are based on all cases with valid data for all variables in the model. |
| Syntax | | GLM na_t1 na_t2 na_t3 BY flowers /WSFACTOR=Stage 3 Simple(1) /MEASURE=Negative_affect /METHOD=SSTYPE(3) /POSTHOC=flowers(BONFERRONI) /EMMEANS=TABLES(flowers*Stage) /PRINT=DESCRIPTIVE ETASQ OPOWER /CRITERIA=ALPHA(.05) /WSDESIGN=Stage /DESIGN=flowers. |
| Resources | Processor Time | 00:00:00.00 |
|  | Elapsed Time | 00:00:00.00 |

| **Within-Subjects Factors** | |
| --- | --- |
| Measure: Negative_affect | |
| Stage | Dependent Variable |
| 1 | na_t1 |
| 2 | na_t2 |
| 3 | na_t3 |

| **Between-Subjects Factors** | | | |
| --- | --- | --- | --- |
|  | | Value Label | N |
| Flowers - The participant mentioned noticing flowers in the comments | 0 | Did not mention flowers | 337 |
|  | 1 | Mentioned flowers | 35 |

| **Descriptive Statistics** | | | | |
| --- | --- | --- | --- | --- |
|  | The participant mentioned noticing flowers in the comments | Mean | Std. Deviation | N |
| Negative Affect baseline score | Did not mention flowers | 7.7567 | 3.55654 | 337 |
|  | Mentioned flowers | 7.6000 | 3.25576 | 35 |
|  | Total | 7.7419 | 3.52550 | 372 |
| Negative Affect score after stressor | Did not mention flowers | 9.4659 | 4.21161 | 337 |
|  | Mentioned flowers | 9.7429 | 4.28639 | 35 |
|  | Total | 9.4919 | 4.21363 | 372 |
| Negative Affect score after video | Did not mention flowers | 6.7122 | 3.15713 | 337 |
|  | Mentioned flowers | 6.1429 | 2.36572 | 35 |
|  | Total | 6.6586 | 3.09318 | 372 |

| **Multivariate Tests**^a^ | | | | | | | | | |
| --- | --- | --- | --- | --- | --- | --- | --- | --- | --- |
| Effect | | Value | F | Hypothesis df | Error df | Sig. | Partial Eta Squared | Noncent. Parameter | Observed Power^c^ |
| Stage | Pillai's Trace | .211 | 49.392^b^ | 2.000 | 369.000 | <.001 | .211 | 98.784 | 1.000 |
|  | Wilks' Lambda | .789 | 49.392^b^ | 2.000 | 369.000 | <.001 | .211 | 98.784 | 1.000 |
|  | Hotelling's Trace | .268 | 49.392^b^ | 2.000 | 369.000 | <.001 | .211 | 98.784 | 1.000 |
|  | Roy's Largest Root | .268 | 49.392^b^ | 2.000 | 369.000 | <.001 | .211 | 98.784 | 1.000 |
| Stage * flowers | Pillai's Trace | .005 | .919^b^ | 2.000 | 369.000 | .400 | .005 | 1.839 | .209 |
|  | Wilks' Lambda | .995 | .919^b^ | 2.000 | 369.000 | .400 | .005 | 1.839 | .209 |
|  | Hotelling's Trace | .005 | .919^b^ | 2.000 | 369.000 | .400 | .005 | 1.839 | .209 |
|  | Roy's Largest Root | .005 | .919^b^ | 2.000 | 369.000 | .400 | .005 | 1.839 | .209 |
| a. Design: Intercept + flowers Within Subjects Design: Stage | | | | | | | | | |
| b. Exact statistic | | | | | | | | | |
| c. Computed using alpha = .05 | | | | | | | | | |

| **Mauchly's Test of Sphericity**^a^ | | | | | | | |
| --- | --- | --- | --- | --- | --- | --- | --- |
| Measure: Negative_affect | | | | | | | |
| Within Subjects Effect | Mauchly's W | Approx. Chi-Square | df | Sig. | Epsilon^b^ | | |
|  |  |  |  |  | Greenhouse-Geisser | Huynh-Feldt | Lower-bound |
| Stage | .890 | 42.968 | 2 | <.001 | .901 | .908 | .500 |
| Tests the null hypothesis that the error covariance matrix of the orthonormalized transformed dependent variables is proportional to an identity matrix. | | | | | | | |
| a. Design: Intercept + flowers Within Subjects Design: Stage | | | | | | | |
| b. May be used to adjust the degrees of freedom for the averaged tests of significance. Corrected tests are displayed in the Tests of Within-Subjects Effects table. | | | | | | | |

| **Tests of Within-Subjects Effects** | | | | | | | | | |
| --- | --- | --- | --- | --- | --- | --- | --- | --- | --- |
| Measure: Negative_affect | | | | | | | | | |
| Source | | Type III Sum of Squares | df | Mean Square | F | Sig. | Partial Eta Squared | Noncent. Parameter | Observed Power^a^ |
| Stage | Sphericity Assumed | 649.636 | 2 | 324.818 | 60.455 | <.001 | .140 | 120.911 | 1.000 |
|  | Greenhouse-Geisser | 649.636 | 1.802 | 360.522 | 60.455 | <.001 | .140 | 108.936 | 1.000 |
|  | Huynh-Feldt | 649.636 | 1.815 | 357.905 | 60.455 | <.001 | .140 | 109.733 | 1.000 |
|  | Lower-bound | 649.636 | 1.000 | 649.636 | 60.455 | <.001 | .140 | 60.455 | 1.000 |
| Stage * flowers | Sphericity Assumed | 11.357 | 2 | 5.678 | 1.057 | .348 | .003 | 2.114 | .236 |
|  | Greenhouse-Geisser | 11.357 | 1.802 | 6.303 | 1.057 | .342 | .003 | 1.904 | .225 |
|  | Huynh-Feldt | 11.357 | 1.815 | 6.257 | 1.057 | .343 | .003 | 1.918 | .225 |
|  | Lower-bound | 11.357 | 1.000 | 11.357 | 1.057 | .305 | .003 | 1.057 | .176 |
| Error(Stage) | Sphericity Assumed | 3975.921 | 740 | 5.373 |  |  |  |  |  |
|  | Greenhouse-Geisser | 3975.921 | 666.715 | 5.963 |  |  |  |  |  |
|  | Huynh-Feldt | 3975.921 | 671.590 | 5.920 |  |  |  |  |  |
|  | Lower-bound | 3975.921 | 370.000 | 10.746 |  |  |  |  |  |
| a. Computed using alpha = .05 | | | | | | | | | |

| **Tests of Within-Subjects Contrasts** | | | | | | | | | |
| --- | --- | --- | --- | --- | --- | --- | --- | --- | --- |
| Measure: Negative_affect | | | | | | | | | |
| Source | Stage | Type III Sum of Squares | df | Mean Square | F | Sig. | Partial Eta Squared | Noncent. Parameter | Observed Power^a^ |
| Stage | Level 2 vs. Level 1 | 470.479 | 1 | 470.479 | 39.475 | <.001 | .096 | 39.475 | 1.000 |
|  | Level 3 vs. Level 1 | 198.431 | 1 | 198.431 | 27.385 | <.001 | .069 | 27.385 | .999 |
| Stage * flowers | Level 2 vs. Level 1 | 5.963 | 1 | 5.963 | .500 | .480 | .001 | .500 | .109 |
|  | Level 3 vs. Level 1 | 5.399 | 1 | 5.399 | .745 | .389 | .002 | .745 | .138 |
| Error(Stage) | Level 2 vs. Level 1 | 4409.787 | 370 | 11.918 |  |  |  |  |  |
|  | Level 3 vs. Level 1 | 2681.018 | 370 | 7.246 |  |  |  |  |  |
| a. Computed using alpha = .05 | | | | | | | | | |

| **Tests of Between-Subjects Effects** | | | | | | | | |
| --- | --- | --- | --- | --- | --- | --- | --- | --- |
| Measure: Negative_affect | | | | | | | | |
| Transformed Variable: Average | | | | | | | | |
| Source | Type III Sum of Squares | df | Mean Square | F | Sig. | Partial Eta Squared | Noncent. Parameter | Observed Power^a^ |
| Intercept | 7922.158 | 1 | 7922.158 | 817.368 | <.001 | .688 | 817.368 | 1.000 |
| flowers | .710 | 1 | .710 | .073 | .787 | .000 | .073 | .058 |
| Error | 3586.145 | 370 | 9.692 |  |  |  |  |  |
| a. Computed using alpha = .05 | | | | | | | | |

**Estimated Marginal Means**

| **Flowers * Stage** | | | | | |
| --- | --- | --- | --- | --- | --- |
| Measure: Negative_affect | | | | | |
| Flowers | Stage | Mean | Std. Error | 95% Confidence Interval | |
|  |  |  |  | Lower Bound | Upper Bound |
| Did not mention flowers | 1 | 7.757 | .192 | 7.379 | 8.135 |
|  | 2 | 9.466 | .230 | 9.014 | 9.918 |
|  | 3 | 6.712 | .168 | 6.381 | 7.043 |
| Mentioned flowers | 1 | 7.600 | .597 | 6.427 | 8.773 |
|  | 2 | 9.743 | .713 | 8.341 | 11.145 |
|  | 3 | 6.143 | .523 | 5.115 | 7.171 |

**Positive Affect**

**General Linear Model**

| **Notes** | | |
| --- | --- | --- |
| Output Created | | 29-MAR-2023 13:59:57 |
| Comments | |  |
| Input | Active Dataset | DataSet1 |
|  | Filter | <none> |
|  | Weight | <none> |
|  | Split File | <none> |
|  | N of Rows in Working Data File | 372 |
| Missing Value Handling | Definition of Missing | User-defined missing values are treated as missing. |
|  | Cases Used | Statistics are based on all cases with valid data for all variables in the model. |
| Syntax | | GLM pa_t1 pa_t2 pa_t3 BY flowers /WSFACTOR=Stage 3 Simple(1) /MEASURE=Positive_affect /METHOD=SSTYPE(3) /POSTHOC=flowers(BONFERRONI) /EMMEANS=TABLES(flowers*Stage) /PRINT=DESCRIPTIVE ETASQ OPOWER /CRITERIA=ALPHA(.05) /WSDESIGN=Stage /DESIGN=flowers. |
| Resources | Processor Time | 00:00:00.00 |
|  | Elapsed Time | 00:00:00.00 |

| **Within-Subjects Factors** | |
| --- | --- |
| Measure: Positive_affect | |
| Stage | Dependent Variable |
| 1 | pa_t1 |
| 2 | pa_t2 |
| 3 | pa_t3 |

| **Between-Subjects Factors** | | | |
| --- | --- | --- | --- |
|  | | Value Label | N |
| Flowers - The participant mentioned noticing flowers in the comments | 0 | Did not mention flowers | 337 |
|  | 1 | Mentioned flowers | 35 |

| **Descriptive Statistics** | | | | |
| --- | --- | --- | --- | --- |
|  | Binary - The participant mentioned noticing flowers in the comments | Mean | Std. Deviation | N |
| Positive Affect baseline score | Did not mention flowers | 14.1424 | 4.12064 | 337 |
|  | Mentioned flowers | 14.4857 | 4.13288 | 35 |
|  | Total | 14.1747 | 4.11743 | 372 |
| Positive Affect score after stressor | Did not mention flowers | 13.6677 | 4.48104 | 337 |
|  | Mentioned flowers | 14.0000 | 4.04388 | 35 |
|  | Total | 13.6989 | 4.43773 | 372 |
| Positive Affect score after video | Did not mention flowers | 13.5846 | 4.58772 | 337 |
|  | Mentioned flowers | 15.6571 | 4.17938 | 35 |
|  | Total | 13.7796 | 4.58579 | 372 |

| **Multivariate Tests**^a^ | | | | | | | | | |
| --- | --- | --- | --- | --- | --- | --- | --- | --- | --- |
| Effect | | Value | F | Hypothesis df | Error df | Sig. | Partial Eta Squared | Noncent. Parameter | Observed Power^c^ |
| Stage | Pillai's Trace | .019 | 3.633^b^ | 2.000 | 369.000 | .027 | .019 | 7.265 | .669 |
|  | Wilks' Lambda | .981 | 3.633^b^ | 2.000 | 369.000 | .027 | .019 | 7.265 | .669 |
|  | Hotelling's Trace | .020 | 3.633^b^ | 2.000 | 369.000 | .027 | .019 | 7.265 | .669 |
|  | Roy's Largest Root | .020 | 3.633^b^ | 2.000 | 369.000 | .027 | .019 | 7.265 | .669 |
| Stage * flowers | Pillai's Trace | .025 | 4.755^b^ | 2.000 | 369.000 | .009 | .025 | 9.510 | .791 |
|  | Wilks' Lambda | .975 | 4.755^b^ | 2.000 | 369.000 | .009 | .025 | 9.510 | .791 |
|  | Hotelling's Trace | .026 | 4.755^b^ | 2.000 | 369.000 | .009 | .025 | 9.510 | .791 |
|  | Roy's Largest Root | .026 | 4.755^b^ | 2.000 | 369.000 | .009 | .025 | 9.510 | .791 |
| a. Design: Intercept + flowers Within Subjects Design: Stage | | | | | | | | | |
| b. Exact statistic | | | | | | | | | |
| c. Computed using alpha = .05 | | | | | | | | | |

| **Mauchly's Test of Sphericity**^a^ | | | | | | | |
| --- | --- | --- | --- | --- | --- | --- | --- |
| Measure: Positive_affect | | | | | | | |
| Within Subjects Effect | Mauchly's W | Approx. Chi-Square | df | Sig. | Epsilon^b^ | | |
|  |  |  |  |  | Greenhouse-Geisser | Huynh-Feldt | Lower-bound |
| Stage | .973 | 10.077 | 2 | .006 | .974 | .981 | .500 |
| Tests the null hypothesis that the error covariance matrix of the orthonormalized transformed dependent variables is proportional to an identity matrix. | | | | | | | |
| a. Design: Intercept + flowers Within Subjects Design: Stage | | | | | | | |
| b. May be used to adjust the degrees of freedom for the averaged tests of significance. Corrected tests are displayed in the Tests of Within-Subjects Effects table. | | | | | | | |

| **Tests of Within-Subjects Effects** | | | | | | | | | |
| --- | --- | --- | --- | --- | --- | --- | --- | --- | --- |
| Measure: Positive_affect | | | | | | | | | |
| Source | | Type III Sum of Squares | df | Mean Square | F | Sig. | Partial Eta Squared | Noncent. Parameter | Observed Power^a^ |
| Stage | Sphericity Assumed | 39.916 | 2 | 19.958 | 3.356 | .035 | .009 | 6.712 | .634 |
|  | Greenhouse-Geisser | 39.916 | 1.948 | 20.495 | 3.356 | .037 | .009 | 6.536 | .626 |
|  | Huynh-Feldt | 39.916 | 1.963 | 20.334 | 3.356 | .036 | .009 | 6.588 | .628 |
|  | Lower-bound | 39.916 | 1.000 | 39.916 | 3.356 | .068 | .009 | 3.356 | .447 |
| Stage * flowers | Sphericity Assumed | 63.614 | 2 | 31.807 | 5.348 | .005 | .014 | 10.697 | .841 |
|  | Greenhouse-Geisser | 63.614 | 1.948 | 32.664 | 5.348 | .005 | .014 | 10.416 | .833 |
|  | Huynh-Feldt | 63.614 | 1.963 | 32.407 | 5.348 | .005 | .014 | 10.499 | .835 |
|  | Lower-bound | 63.614 | 1.000 | 63.614 | 5.348 | .021 | .014 | 5.348 | .636 |
| Error(Stage) | Sphericity Assumed | 4400.810 | 740 | 5.947 |  |  |  |  |  |
|  | Greenhouse-Geisser | 4400.810 | 720.588 | 6.107 |  |  |  |  |  |
|  | Huynh-Feldt | 4400.810 | 726.306 | 6.059 |  |  |  |  |  |
|  | Lower-bound | 4400.810 | 370.000 | 11.894 |  |  |  |  |  |
| a. Computed using alpha = .05 | | | | | | | | | |

| **Tests of Within-Subjects Contrasts** | | | | | | | | | |
| --- | --- | --- | --- | --- | --- | --- | --- | --- | --- |
| Measure: Positive_affect | | | | | | | | | |
| Source | Stage | Type III Sum of Squares | df | Mean Square | F | Sig. | Partial Eta Squared | Noncent. Parameter | Observed Power^a^ |
| Stage | Level 2 vs. Level 1 | 29.251 | 1 | 29.251 | 2.839 | .093 | .008 | 2.839 | .390 |
|  | Level 3 vs. Level 1 | 11.936 | 1 | 11.936 | .873 | .351 | .002 | .873 | .154 |
| Stage * flowers | Level 2 vs. Level 1 | .004 | 1 | .004 | .000 | .985 | .000 | .000 | .050 |
|  | Level 3 vs. Level 1 | 94.818 | 1 | 94.818 | 6.936 | .009 | .018 | 6.936 | .748 |
| Error(Stage) | Level 2 vs. Level 1 | 3812.778 | 370 | 10.305 |  |  |  |  |  |
|  | Level 3 vs. Level 1 | 5058.093 | 370 | 13.671 |  |  |  |  |  |
| a. Computed using alpha = .05 | | | | | | | | | |

| **Tests of Between-Subjects Effects** | | | | | | | | |
| --- | --- | --- | --- | --- | --- | --- | --- | --- |
| Measure: Positive_affect | | | | | | | | |
| Transformed Variable: Average | | | | | | | | |
| Source | Type III Sum of Squares | df | Mean Square | F | Sig. | Partial Eta Squared | Noncent. Parameter | Observed Power^a^ |
| Intercept | 25776.608 | 1 | 25776.608 | 1697.681 | <.001 | .821 | 1697.681 | 1.000 |
| flowers | 26.608 | 1 | 26.608 | 1.752 | .186 | .005 | 1.752 | .262 |
| Error | 5617.866 | 370 | 15.183 |  |  |  |  |  |
| a. Computed using alpha = .05 | | | | | | | | |

**Estimated Marginal Means**

| **Flowers * Stage** | | | | | |
| --- | --- | --- | --- | --- | --- |
| Measure: Positive_affect | | | | | |
| Flowers | Stage | Mean | Std. Error | 95% Confidence Interval | |
|  |  |  |  | Lower Bound | Upper Bound |
| Did not mention flowers | 1 | 14.142 | .225 | 13.701 | 14.584 |
|  | 2 | 13.668 | .242 | 13.192 | 14.144 |
|  | 3 | 13.585 | .248 | 13.097 | 14.072 |
| Mentioned flowers | 1 | 14.486 | .697 | 13.116 | 15.856 |
|  | 2 | 14.000 | .751 | 12.523 | 15.477 |
|  | 3 | 15.657 | .769 | 14.144 | 17.170 |

**Noticing Sounds**

**Negative Affect**

**General Linear Model**

| **Notes** | | |
| --- | --- | --- |
| Output Created | | 29-MAR-2023 14:03:25 |
| Comments | |  |
| Input | Active Dataset | DataSet1 |
|  | Filter | <none> |
|  | Weight | <none> |
|  | Split File | <none> |
|  | N of Rows in Working Data File | 372 |
| Missing Value Handling | Definition of Missing | User-defined missing values are treated as missing. |
|  | Cases Used | Statistics are based on all cases with valid data for all variables in the model. |
| Syntax | | GLM na_t1 na_t2 na_t3 BY sounds /WSFACTOR=Stage 3 Simple(1) /MEASURE=Negative_affect /METHOD=SSTYPE(3) /POSTHOC=sounds(BONFERRONI) /EMMEANS=TABLES(sounds) /PRINT=DESCRIPTIVE ETASQ OPOWER /CRITERIA=ALPHA(.05) /WSDESIGN=Stage /DESIGN=sounds. |
| Resources | Processor Time | 00:00:00.00 |
|  | Elapsed Time | 00:00:00.00 |

| **Within-Subjects Factors** | |
| --- | --- |
| Measure: Negative_affect | |
| Stage | Dependent Variable |
| 1 | na_t1 |
| 2 | na_t2 |
| 3 | na_t3 |

| **Between-Subjects Factors** | | | |
| --- | --- | --- | --- |
|  | | Value Label | N |
| Sounds - The participant mentioned noticing sounds in the comments | 0 | no comment | 317 |
|  | 1 | mentioned sounds | 55 |

| **Descriptive Statistics** | | | | |
| --- | --- | --- | --- | --- |
|  | Binary - The participant mentioned noticing sounds in the comments | Mean | Std. Deviation | N |
| Negative Affect baseline score | no comment | 7.9306 | 3.66754 | 317 |
|  | mentioned sounds | 6.6545 | 2.29507 | 55 |
|  | Total | 7.7419 | 3.52550 | 372 |
| Negative Affect score after stressor | no comment | 9.6372 | 4.37169 | 317 |
|  | mentioned sounds | 8.6545 | 3.05031 | 55 |
|  | Total | 9.4919 | 4.21363 | 372 |
| Negative Affect score after video | no comment | 6.8391 | 3.27282 | 317 |
|  | mentioned sounds | 5.6182 | 1.32624 | 55 |
|  | Total | 6.6586 | 3.09318 | 372 |

| **Multivariate Tests**^a^ | | | | | | | | | |
| --- | --- | --- | --- | --- | --- | --- | --- | --- | --- |
| Effect | | Value | F | Hypothesis df | Error df | Sig. | Partial Eta Squared | Noncent. Parameter | Observed Power^c^ |
| Stage | Pillai's Trace | .248 | 60.847^b^ | 2.000 | 369.000 | <.001 | .248 | 121.694 | 1.000 |
|  | Wilks' Lambda | .752 | 60.847^b^ | 2.000 | 369.000 | <.001 | .248 | 121.694 | 1.000 |
|  | Hotelling's Trace | .330 | 60.847^b^ | 2.000 | 369.000 | <.001 | .248 | 121.694 | 1.000 |
|  | Roy's Largest Root | .330 | 60.847^b^ | 2.000 | 369.000 | <.001 | .248 | 121.694 | 1.000 |
| Stage * sounds | Pillai's Trace | .001 | .170^b^ | 2.000 | 369.000 | .844 | .001 | .340 | .076 |
|  | Wilks' Lambda | .999 | .170^b^ | 2.000 | 369.000 | .844 | .001 | .340 | .076 |
|  | Hotelling's Trace | .001 | .170^b^ | 2.000 | 369.000 | .844 | .001 | .340 | .076 |
|  | Roy's Largest Root | .001 | .170^b^ | 2.000 | 369.000 | .844 | .001 | .340 | .076 |
| a. Design: Intercept + sounds Within Subjects Design: Stage | | | | | | | | | |
| b. Exact statistic | | | | | | | | | |
| c. Computed using alpha = .05 | | | | | | | | | |

| **Mauchly's Test of Sphericity**^a^ | | | | | | | |
| --- | --- | --- | --- | --- | --- | --- | --- |
| Measure: Negative_affect | | | | | | | |
| Within Subjects Effect | Mauchly's W | Approx. Chi-Square | df | Sig. | Epsilon^b^ | | |
|  |  |  |  |  | Greenhouse-Geisser | Huynh-Feldt | Lower-bound |
| Stage | .890 | 43.157 | 2 | <.001 | .901 | .907 | .500 |
| Tests the null hypothesis that the error covariance matrix of the orthonormalized transformed dependent variables is proportional to an identity matrix. | | | | | | | |
| a. Design: Intercept + sounds Within Subjects Design: Stage | | | | | | | |
| b. May be used to adjust the degrees of freedom for the averaged tests of significance. Corrected tests are displayed in the Tests of Within-Subjects Effects table. | | | | | | | |

| **Tests of Within-Subjects Effects** | | | | | | | | | |
| --- | --- | --- | --- | --- | --- | --- | --- | --- | --- |
| Measure: Negative_affect | | | | | | | | | |
| Source | | Type III Sum of Squares | df | Mean Square | F | Sig. | Partial Eta Squared | Noncent. Parameter | Observed Power^a^ |
| Stage | Sphericity Assumed | 817.193 | 2 | 408.596 | 75.875 | <.001 | .170 | 151.750 | 1.000 |
|  | Greenhouse-Geisser | 817.193 | 1.801 | 453.695 | 75.875 | <.001 | .170 | 136.665 | 1.000 |
|  | Huynh-Feldt | 817.193 | 1.814 | 450.404 | 75.875 | <.001 | .170 | 137.664 | 1.000 |
|  | Lower-bound | 817.193 | 1.000 | 817.193 | 75.875 | <.001 | .170 | 75.875 | 1.000 |
| Stage * sounds | Sphericity Assumed | 2.279 | 2 | 1.139 | .212 | .809 | .001 | .423 | .083 |
|  | Greenhouse-Geisser | 2.279 | 1.801 | 1.265 | .212 | .786 | .001 | .381 | .082 |
|  | Huynh-Feldt | 2.279 | 1.814 | 1.256 | .212 | .788 | .001 | .384 | .082 |
|  | Lower-bound | 2.279 | 1.000 | 2.279 | .212 | .646 | .001 | .212 | .074 |
| Error(Stage) | Sphericity Assumed | 3984.999 | 740 | 5.385 |  |  |  |  |  |
|  | Greenhouse-Geisser | 3984.999 | 666.441 | 5.980 |  |  |  |  |  |
|  | Huynh-Feldt | 3984.999 | 671.312 | 5.936 |  |  |  |  |  |
|  | Lower-bound | 3984.999 | 370.000 | 10.770 |  |  |  |  |  |
| a. Computed using alpha = .05 | | | | | | | | | |

| **Tests of Within-Subjects Contrasts** | | | | | | | | | |
| --- | --- | --- | --- | --- | --- | --- | --- | --- | --- |
| Measure: Negative_affect | | | | | | | | | |
| Source | Stage | Type III Sum of Squares | df | Mean Square | F | Sig. | Partial Eta Squared | Noncent. Parameter | Observed Power^a^ |
| Stage | Level 2 vs. Level 1 | 643.926 | 1 | 643.926 | 54.005 | <.001 | .127 | 54.005 | 1.000 |
|  | Level 3 vs. Level 1 | 212.207 | 1 | 212.207 | 29.229 | <.001 | .073 | 29.229 | 1.000 |
| Stage * sounds | Level 2 vs. Level 1 | 4.034 | 1 | 4.034 | .338 | .561 | .001 | .338 | .089 |
|  | Level 3 vs. Level 1 | .142 | 1 | .142 | .020 | .889 | .000 | .020 | .052 |
| Error(Stage) | Level 2 vs. Level 1 | 4411.716 | 370 | 11.924 |  |  |  |  |  |
|  | Level 3 vs. Level 1 | 2686.274 | 370 | 7.260 |  |  |  |  |  |
| a. Computed using alpha = .05 | | | | | | | | | |

| **Tests of Between-Subjects Effects** | | | | | | | | |
| --- | --- | --- | --- | --- | --- | --- | --- | --- |
| Measure: Negative_affect | | | | | | | | |
| Transformed Variable: Average | | | | | | | | |
| Source | Type III Sum of Squares | df | Mean Square | F | Sig. | Partial Eta Squared | Noncent. Parameter | Observed Power^a^ |
| Intercept | 10702.584 | 1 | 10702.584 | 1123.774 | <.001 | .752 | 1123.774 | 1.000 |
| sounds | 63.054 | 1 | 63.054 | 6.621 | .010 | .018 | 6.621 | .728 |
| Error | 3523.802 | 370 | 9.524 |  |  |  |  |  |
| a. Computed using alpha = .05 | | | | | | | | |

**Estimated Marginal Means**

| **Sounds - The participant mentioned noticing sounds in the comments** | | | | |
| --- | --- | --- | --- | --- |
| Measure: Negative_affect | | | | |
| Binary - The participant mentioned noticing sounds in the comments | Mean | Std. Error | 95% Confidence Interval | |
|  |  |  | Lower Bound | Upper Bound |
| no comment | 8.136 | .173 | 7.795 | 8.476 |
| mentioned sounds | 6.976 | .416 | 6.157 | 7.794 |

**Positive Affect**

**General Linear Model**

| **Notes** | | |
| --- | --- | --- |
| Output Created | | 29-MAR-2023 14:04:35 |
| Comments | |  |
| Input | Active Dataset | DataSet1 |
|  | Filter | <none> |
|  | Weight | <none> |
|  | Split File | <none> |
|  | N of Rows in Working Data File | 372 |
| Missing Value Handling | Definition of Missing | User-defined missing values are treated as missing. |
|  | Cases Used | Statistics are based on all cases with valid data for all variables in the model. |
| Syntax | | GLM pa_t1 pa_t2 pa_t3 BY sounds /WSFACTOR=Stage 3 Simple(1) /MEASURE=Positive_affect /METHOD=SSTYPE(3) /POSTHOC=sounds(BONFERRONI) /EMMEANS=TABLES(sounds) /PRINT=DESCRIPTIVE ETASQ OPOWER /CRITERIA=ALPHA(.05) /WSDESIGN=Stage /DESIGN=sounds. |
| Resources | Processor Time | 00:00:00.00 |
|  | Elapsed Time | 00:00:00.00 |

| **Within-Subjects Factors** | |
| --- | --- |
| Measure: Positive_affect | |
| Stage | Dependent Variable |
| 1 | pa_t1 |
| 2 | pa_t2 |
| 3 | pa_t3 |

| **Between-Subjects Factors** | | | |
| --- | --- | --- | --- |
|  | | Value Label | N |
| Binary - The participant mentioned noticing sounds in the comments | 0 | no comment | 317 |
|  | 1 | mentioned sounds | 55 |

| **Descriptive Statistics** | | | | |
| --- | --- | --- | --- | --- |
|  | Binary - The participant mentioned noticing sounds in the comments | Mean | Std. Deviation | N |
| Positive Affect baseline score | no comment | 14.0789 | 4.16701 | 317 |
|  | mentioned sounds | 14.7273 | 3.80767 | 55 |
|  | Total | 14.1747 | 4.11743 | 372 |
| Positive Affect score after stressor | no comment | 13.6246 | 4.46659 | 317 |
|  | mentioned sounds | 14.1273 | 4.28198 | 55 |
|  | Total | 13.6989 | 4.43773 | 372 |
| Positive Affect score after video | no comment | 13.5489 | 4.46877 | 317 |
|  | mentioned sounds | 15.1091 | 5.05039 | 55 |
|  | Total | 13.7796 | 4.58579 | 372 |

| **Multivariate Tests**^a^ | | | | | | | | | |
| --- | --- | --- | --- | --- | --- | --- | --- | --- | --- |
| Effect | | Value | F | Hypothesis df | Error df | Sig. | Partial Eta Squared | Noncent. Parameter | Observed Power^c^ |
| Stage | Pillai's Trace | .016 | 3.054^b^ | 2.000 | 369.000 | .048 | .016 | 6.109 | .589 |
|  | Wilks' Lambda | .984 | 3.054^b^ | 2.000 | 369.000 | .048 | .016 | 6.109 | .589 |
|  | Hotelling's Trace | .017 | 3.054^b^ | 2.000 | 369.000 | .048 | .016 | 6.109 | .589 |
|  | Roy's Largest Root | .017 | 3.054^b^ | 2.000 | 369.000 | .048 | .016 | 6.109 | .589 |
| Stage * sounds | Pillai's Trace | .013 | 2.345^b^ | 2.000 | 369.000 | .097 | .013 | 4.690 | .474 |
|  | Wilks' Lambda | .987 | 2.345^b^ | 2.000 | 369.000 | .097 | .013 | 4.690 | .474 |
|  | Hotelling's Trace | .013 | 2.345^b^ | 2.000 | 369.000 | .097 | .013 | 4.690 | .474 |
|  | Roy's Largest Root | .013 | 2.345^b^ | 2.000 | 369.000 | .097 | .013 | 4.690 | .474 |
| a. Design: Intercept + sounds Within Subjects Design: Stage | | | | | | | | | |
| b. Exact statistic | | | | | | | | | |
| c. Computed using alpha = .05 | | | | | | | | | |

| **Mauchly's Test of Sphericity**^a^ | | | | | | | |
| --- | --- | --- | --- | --- | --- | --- | --- |
| Measure: Positive_affect | | | | | | | |
| Within Subjects Effect | Mauchly's W | Approx. Chi-Square | df | Sig. | Epsilon^b^ | | |
|  |  |  |  |  | Greenhouse-Geisser | Huynh-Feldt | Lower-bound |
| Stage | .971 | 10.832 | 2 | .004 | .972 | .980 | .500 |
| Tests the null hypothesis that the error covariance matrix of the orthonormalized transformed dependent variables is proportional to an identity matrix. | | | | | | | |
| a. Design: Intercept + sounds Within Subjects Design: Stage | | | | | | | |
| b. May be used to adjust the degrees of freedom for the averaged tests of significance. Corrected tests are displayed in the Tests of Within-Subjects Effects table. | | | | | | | |

| **Tests of Within-Subjects Effects** | | | | | | | | | |
| --- | --- | --- | --- | --- | --- | --- | --- | --- | --- |
| Measure: Positive_affect | | | | | | | | | |
| Source | | Type III Sum of Squares | df | Mean Square | F | Sig. | Partial Eta Squared | Noncent. Parameter | Observed Power^a^ |
| Stage | Sphericity Assumed | 30.534 | 2 | 15.267 | 2.548 | .079 | .007 | 5.096 | .510 |
|  | Greenhouse-Geisser | 30.534 | 1.944 | 15.709 | 2.548 | .081 | .007 | 4.953 | .502 |
|  | Huynh-Feldt | 30.534 | 1.959 | 15.585 | 2.548 | .080 | .007 | 4.992 | .504 |
|  | Lower-bound | 30.534 | 1.000 | 30.534 | 2.548 | .111 | .007 | 2.548 | .357 |
| Stage * sounds | Sphericity Assumed | 30.792 | 2 | 15.396 | 2.570 | .077 | .007 | 5.139 | .514 |
|  | Greenhouse-Geisser | 30.792 | 1.944 | 15.841 | 2.570 | .079 | .007 | 4.995 | .506 |
|  | Huynh-Feldt | 30.792 | 1.959 | 15.717 | 2.570 | .078 | .007 | 5.034 | .508 |
|  | Lower-bound | 30.792 | 1.000 | 30.792 | 2.570 | .110 | .007 | 2.570 | .359 |
| Error(Stage) | Sphericity Assumed | 4433.633 | 740 | 5.991 |  |  |  |  |  |
|  | Greenhouse-Geisser | 4433.633 | 719.196 | 6.165 |  |  |  |  |  |
|  | Huynh-Feldt | 4433.633 | 724.891 | 6.116 |  |  |  |  |  |
|  | Lower-bound | 4433.633 | 370.000 | 11.983 |  |  |  |  |  |
| a. Computed using alpha = .05 | | | | | | | | | |

| **Tests of Within-Subjects Contrasts** | | | | | | | | | |
| --- | --- | --- | --- | --- | --- | --- | --- | --- | --- |
| Measure: Positive_affect | | | | | | | | | |
| Source | Stage | Type III Sum of Squares | df | Mean Square | F | Sig. | Partial Eta Squared | Noncent. Parameter | Observed Power^a^ |
| Stage | Level 2 vs. Level 1 | 52.092 | 1 | 52.092 | 5.056 | .025 | .013 | 5.056 | .611 |
|  | Level 3 vs. Level 1 | 1.029 | 1 | 1.029 | .074 | .785 | .000 | .074 | .059 |
| Stage * sounds | Level 2 vs. Level 1 | .996 | 1 | .996 | .097 | .756 | .000 | .097 | .061 |
|  | Level 3 vs. Level 1 | 38.964 | 1 | 38.964 | 2.819 | .094 | .008 | 2.819 | .388 |
| Error(Stage) | Level 2 vs. Level 1 | 3811.787 | 370 | 10.302 |  |  |  |  |  |
|  | Level 3 vs. Level 1 | 5113.947 | 370 | 13.821 |  |  |  |  |  |
| a. Computed using alpha = .05 | | | | | | | | | |

| **Tests of Between-Subjects Effects** | | | | | | | | |
| --- | --- | --- | --- | --- | --- | --- | --- | --- |
| Measure: Positive_affect | | | | | | | | |
| Transformed Variable: Average | | | | | | | | |
| Source | Type III Sum of Squares | df | Mean Square | F | Sig. | Partial Eta Squared | Noncent. Parameter | Observed Power^a^ |
| Intercept | 37816.281 | 1 | 37816.281 | 2495.816 | <.001 | .871 | 2495.816 | 1.000 |
| sounds | 38.281 | 1 | 38.281 | 2.526 | .113 | .007 | 2.526 | .354 |
| Error | 5606.193 | 370 | 15.152 |  |  |  |  |  |
| a. Computed using alpha = .05 | | | | | | | | |

**Estimated Marginal Means**

| **Sounds - The participant mentioned noticing sounds in the comments** | | | | |
| --- | --- | --- | --- | --- |
| Measure: Positive_affect | | | | |
| Binary - The participant mentioned noticing sounds in the comments | Mean | Std. Error | 95% Confidence Interval | |
|  |  |  | Lower Bound | Upper Bound |
| no comment | 13.751 | .219 | 13.321 | 14.181 |
| mentioned sounds | 14.655 | .525 | 13.622 | 15.687 |

**Noticing Trees**

**Negative Affect**

**General Linear Model**

| **Notes** | | |
| --- | --- | --- |
| Output Created | | 29-MAR-2023 14:06:09 |
| Comments | |  |
| Input | Active Dataset | DataSet1 |
|  | Filter | <none> |
|  | Weight | <none> |
|  | Split File | <none> |
|  | N of Rows in Working Data File | 372 |
| Missing Value Handling | Definition of Missing | User-defined missing values are treated as missing. |
|  | Cases Used | Statistics are based on all cases with valid data for all variables in the model. |
| Syntax | | GLM na_t1 na_t2 na_t3 BY trees /WSFACTOR=Stage 3 Simple(1) /MEASURE=Negative_affect /METHOD=SSTYPE(3) /EMMEANS=TABLES(trees*Stage) /PRINT=DESCRIPTIVE ETASQ OPOWER /CRITERIA=ALPHA(.05) /WSDESIGN=Stage /DESIGN=trees. |
| Resources | Processor Time | 00:00:00.00 |
|  | Elapsed Time | 00:00:00.00 |

| **Within-Subjects Factors** | |
| --- | --- |
| Measure: Negative_affect | |
| Stage | Dependent Variable |
| 1 | na_t1 |
| 2 | na_t2 |
| 3 | na_t3 |

| **Between-Subjects Factors** | | | |
| --- | --- | --- | --- |
|  | | Value Label | N |
| Trees - The participant mentioned noticing trees in the comments | 0 | no comments | 338 |
|  | 1 | noticed trees | 34 |

| **Descriptive Statistics** | | | | |
| --- | --- | --- | --- | --- |
|  | Binary - The participant mentioned noticing trees in the comments | Mean | Std. Deviation | N |
| Negative Affect baseline score | no comments | 7.8254 | 3.60049 | 338 |
|  | noticed trees | 6.9118 | 2.56276 | 34 |
|  | Total | 7.7419 | 3.52550 | 372 |
| Negative Affect score after stressor | no comments | 9.6036 | 4.29784 | 338 |
|  | noticed trees | 8.3824 | 3.09462 | 34 |
|  | Total | 9.4919 | 4.21363 | 372 |
| Negative Affect score after video | no comments | 6.7633 | 3.21027 | 338 |
|  | noticed trees | 5.6176 | 1.04489 | 34 |
|  | Total | 6.6586 | 3.09318 | 372 |

| **Multivariate Tests**^a^ | | | | | | | | | |
| --- | --- | --- | --- | --- | --- | --- | --- | --- | --- |
| Effect | | Value | F | Hypothesis df | Error df | Sig. | Partial Eta Squared | Noncent. Parameter | Observed Power^c^ |
| Stage | Pillai's Trace | .169 | 37.639^b^ | 2.000 | 369.000 | <.001 | .169 | 75.277 | 1.000 |
|  | Wilks' Lambda | .831 | 37.639^b^ | 2.000 | 369.000 | <.001 | .169 | 75.277 | 1.000 |
|  | Hotelling's Trace | .204 | 37.639^b^ | 2.000 | 369.000 | <.001 | .169 | 75.277 | 1.000 |
|  | Roy's Largest Root | .204 | 37.639^b^ | 2.000 | 369.000 | <.001 | .169 | 75.277 | 1.000 |
| Stage * trees | Pillai's Trace | .001 | .178^b^ | 2.000 | 369.000 | .837 | .001 | .357 | .078 |
|  | Wilks' Lambda | .999 | .178^b^ | 2.000 | 369.000 | .837 | .001 | .357 | .078 |
|  | Hotelling's Trace | .001 | .178^b^ | 2.000 | 369.000 | .837 | .001 | .357 | .078 |
|  | Roy's Largest Root | .001 | .178^b^ | 2.000 | 369.000 | .837 | .001 | .357 | .078 |
| a. Design: Intercept + trees Within Subjects Design: Stage | | | | | | | | | |
| b. Exact statistic | | | | | | | | | |
| c. Computed using alpha = .05 | | | | | | | | | |

| **Mauchly's Test of Sphericity**^a^ | | | | | | | |
| --- | --- | --- | --- | --- | --- | --- | --- |
| Measure: Negative_affect | | | | | | | |
| Within Subjects Effect | Mauchly's W | Approx. Chi-Square | df | Sig. | Epsilon^b^ | | |
|  |  |  |  |  | Greenhouse-Geisser | Huynh-Feldt | Lower-bound |
| Stage | .889 | 43.301 | 2 | <.001 | .900 | .907 | .500 |
| Tests the null hypothesis that the error covariance matrix of the orthonormalized transformed dependent variables is proportional to an identity matrix. | | | | | | | |
| a. Design: Intercept + trees Within Subjects Design: Stage | | | | | | | |
| b. May be used to adjust the degrees of freedom for the averaged tests of significance. Corrected tests are displayed in the Tests of Within-Subjects Effects table. | | | | | | | |

| **Tests of Within-Subjects Effects** | | | | | | | | | |
| --- | --- | --- | --- | --- | --- | --- | --- | --- | --- |
| Measure: Negative_affect | | | | | | | | | |
| Source | | Type III Sum of Squares | df | Mean Square | F | Sig. | Partial Eta Squared | Noncent. Parameter | Observed Power^a^ |
| Stage | Sphericity Assumed | 489.350 | 2 | 244.675 | 45.427 | <.001 | .109 | 90.855 | 1.000 |
|  | Greenhouse-Geisser | 489.350 | 1.801 | 271.767 | 45.427 | <.001 | .109 | 81.798 | 1.000 |
|  | Huynh-Feldt | 489.350 | 1.814 | 269.796 | 45.427 | <.001 | .109 | 82.395 | 1.000 |
|  | Lower-bound | 489.350 | 1.000 | 489.350 | 45.427 | <.001 | .109 | 45.427 | 1.000 |
| Stage * trees | Sphericity Assumed | 1.587 | 2 | .793 | .147 | .863 | .000 | .295 | .073 |
|  | Greenhouse-Geisser | 1.587 | 1.801 | .881 | .147 | .842 | .000 | .265 | .072 |
|  | Huynh-Feldt | 1.587 | 1.814 | .875 | .147 | .843 | .000 | .267 | .072 |
|  | Lower-bound | 1.587 | 1.000 | 1.587 | .147 | .701 | .000 | .147 | .067 |
| Error(Stage) | Sphericity Assumed | 3985.691 | 740 | 5.386 |  |  |  |  |  |
|  | Greenhouse-Geisser | 3985.691 | 666.232 | 5.982 |  |  |  |  |  |
|  | Huynh-Feldt | 3985.691 | 671.099 | 5.939 |  |  |  |  |  |
|  | Lower-bound | 3985.691 | 370.000 | 10.772 |  |  |  |  |  |
| a. Computed using alpha = .05 | | | | | | | | | |

| **Tests of Within-Subjects Contrasts** | | | | | | | | | |
| --- | --- | --- | --- | --- | --- | --- | --- | --- | --- |
| Measure: Negative_affect | | | | | | | | | |
| Source | Stage | Type III Sum of Squares | df | Mean Square | F | Sig. | Partial Eta Squared | Noncent. Parameter | Observed Power^a^ |
| Stage | Level 2 vs. Level 1 | 326.040 | 1 | 326.040 | 27.337 | <.001 | .069 | 27.337 | .999 |
|  | Level 3 vs. Level 1 | 171.512 | 1 | 171.512 | 23.637 | <.001 | .060 | 23.637 | .998 |
| Stage * trees | Level 2 vs. Level 1 | 2.921 | 1 | 2.921 | .245 | .621 | .001 | .245 | .078 |
|  | Level 3 vs. Level 1 | 1.663 | 1 | 1.663 | .229 | .632 | .001 | .229 | .076 |
| Error(Stage) | Level 2 vs. Level 1 | 4412.829 | 370 | 11.927 |  |  |  |  |  |
|  | Level 3 vs. Level 1 | 2684.754 | 370 | 7.256 |  |  |  |  |  |
| a. Computed using alpha = .05 | | | | | | | | | |

| **Tests of Between-Subjects Effects** | | | | | | | | |
| --- | --- | --- | --- | --- | --- | --- | --- | --- |
| Measure: Negative_affect | | | | | | | | |
| Transformed Variable: Average | | | | | | | | |
| Source | Type III Sum of Squares | df | Mean Square | F | Sig. | Partial Eta Squared | Noncent. Parameter | Observed Power^a^ |
| Intercept | 6982.994 | 1 | 6982.994 | 727.822 | <.001 | .663 | 727.822 | 1.000 |
| trees | 36.940 | 1 | 36.940 | 3.850 | .050 | .010 | 3.850 | .499 |
| Error | 3549.915 | 370 | 9.594 |  |  |  |  |  |
| a. Computed using alpha = .05 | | | | | | | | |

**Estimated Marginal Means**

| **Trees * Stage** | | | | | |
| --- | --- | --- | --- | --- | --- |
| Measure: Negative_affect | | | | | |
| Binary - The participant mentioned noticing trees in the comments | Stage | Mean | Std. Error | 95% Confidence Interval | |
|  |  |  |  | Lower Bound | Upper Bound |
| no comments | 1 | 7.825 | .191 | 7.449 | 8.202 |
|  | 2 | 9.604 | .229 | 9.154 | 10.053 |
|  | 3 | 6.763 | .168 | 6.434 | 7.093 |
| noticed trees | 1 | 6.912 | .604 | 5.725 | 8.099 |
|  | 2 | 8.382 | .721 | 6.964 | 9.800 |
|  | 3 | 5.618 | .528 | 4.579 | 6.656 |

**Positive Affect**

**General Linear Model**

| **Notes** | | |
| --- | --- | --- |
| Output Created | | 29-MAR-2023 14:07:46 |
| Comments | |  |
| Input | Active Dataset | DataSet1 |
|  | Filter | <none> |
|  | Weight | <none> |
|  | Split File | <none> |
|  | N of Rows in Working Data File | 372 |
| Missing Value Handling | Definition of Missing | User-defined missing values are treated as missing. |
|  | Cases Used | Statistics are based on all cases with valid data for all variables in the model. |
| Syntax | | GLM pa_t1 pa_t2 pa_t3 BY trees /WSFACTOR=Stage 3 Simple(1) /MEASURE=Positive_affect /METHOD=SSTYPE(3) /EMMEANS=TABLES(trees*Stage) /PRINT=DESCRIPTIVE ETASQ OPOWER /CRITERIA=ALPHA(.05) /WSDESIGN=Stage /DESIGN=trees. |
| Resources | Processor Time | 00:00:00.00 |
|  | Elapsed Time | 00:00:00.00 |

| **Within-Subjects Factors** | |
| --- | --- |
| Measure: Positive_affect | |
| Stage | Dependent Variable |
| 1 | pa_t1 |
| 2 | pa_t2 |
| 3 | pa_t3 |

| **Between-Subjects Factors** | | | |
| --- | --- | --- | --- |
|  | | Value Label | N |
| Binary - The participant mentioned noticing trees in the comments | 0 | no comments | 338 |
|  | 1 | noticed trees | 34 |

| **Descriptive Statistics** | | | | |
| --- | --- | --- | --- | --- |
|  | Binary - The participant mentioned noticing trees in the comments | Mean | Std. Deviation | N |
| Positive Affect baseline score | no comments | 14.3136 | 4.07306 | 338 |
|  | noticed trees | 12.7941 | 4.36084 | 34 |
|  | Total | 14.1747 | 4.11743 | 372 |
| Positive Affect score after stressor | no comments | 13.8166 | 4.46172 | 338 |
|  | noticed trees | 12.5294 | 4.06936 | 34 |
|  | Total | 13.6989 | 4.43773 | 372 |
| Positive Affect score after video | no comments | 13.7633 | 4.59617 | 338 |
|  | noticed trees | 13.9412 | 4.54567 | 34 |
|  | Total | 13.7796 | 4.58579 | 372 |

| **Multivariate Tests**^a^ | | | | | | | | | |
| --- | --- | --- | --- | --- | --- | --- | --- | --- | --- |
| Effect | | Value | F | Hypothesis df | Error df | Sig. | Partial Eta Squared | Noncent. Parameter | Observed Power^c^ |
| Stage | Pillai's Trace | .014 | 2.547^b^ | 2.000 | 369.000 | .080 | .014 | 5.094 | .508 |
|  | Wilks' Lambda | .986 | 2.547^b^ | 2.000 | 369.000 | .080 | .014 | 5.094 | .508 |
|  | Hotelling's Trace | .014 | 2.547^b^ | 2.000 | 369.000 | .080 | .014 | 5.094 | .508 |
|  | Roy's Largest Root | .014 | 2.547^b^ | 2.000 | 369.000 | .080 | .014 | 5.094 | .508 |
| Stage * trees | Pillai's Trace | .020 | 3.801^b^ | 2.000 | 369.000 | .023 | .020 | 7.602 | .690 |
|  | Wilks' Lambda | .980 | 3.801^b^ | 2.000 | 369.000 | .023 | .020 | 7.602 | .690 |
|  | Hotelling's Trace | .021 | 3.801^b^ | 2.000 | 369.000 | .023 | .020 | 7.602 | .690 |
|  | Roy's Largest Root | .021 | 3.801^b^ | 2.000 | 369.000 | .023 | .020 | 7.602 | .690 |
| a. Design: Intercept + trees Within Subjects Design: Stage | | | | | | | | | |
| b. Exact statistic | | | | | | | | | |
| c. Computed using alpha = .05 | | | | | | | | | |

| **Mauchly's Test of Sphericity**^a^ | | | | | | | |
| --- | --- | --- | --- | --- | --- | --- | --- |
| Measure: Positive_affect | | | | | | | |
| Within Subjects Effect | Mauchly's W | Approx. Chi-Square | df | Sig. | Epsilon^b^ | | |
|  |  |  |  |  | Greenhouse-Geisser | Huynh-Feldt | Lower-bound |
| Stage | .973 | 10.103 | 2 | .006 | .974 | .981 | .500 |
| Tests the null hypothesis that the error covariance matrix of the orthonormalized transformed dependent variables is proportional to an identity matrix. | | | | | | | |
| a. Design: Intercept + trees Within Subjects Design: Stage | | | | | | | |
| b. May be used to adjust the degrees of freedom for the averaged tests of significance. Corrected tests are displayed in the Tests of Within-Subjects Effects table. | | | | | | | |

| **Tests of Within-Subjects Effects** | | | | | | | | | |
| --- | --- | --- | --- | --- | --- | --- | --- | --- | --- |
| Measure: Positive_affect | | | | | | | | | |
| Source | | Type III Sum of Squares | df | Mean Square | F | Sig. | Partial Eta Squared | Noncent. Parameter | Observed Power^a^ |
| Stage | Sphericity Assumed | 28.647 | 2 | 14.323 | 2.402 | .091 | .006 | 4.805 | .485 |
|  | Greenhouse-Geisser | 28.647 | 1.947 | 14.710 | 2.402 | .093 | .006 | 4.678 | .478 |
|  | Huynh-Feldt | 28.647 | 1.963 | 14.595 | 2.402 | .092 | .006 | 4.715 | .480 |
|  | Lower-bound | 28.647 | 1.000 | 28.647 | 2.402 | .122 | .006 | 2.402 | .340 |
| Stage * trees | Sphericity Assumed | 52.324 | 2 | 26.162 | 4.388 | .013 | .012 | 8.776 | .758 |
|  | Greenhouse-Geisser | 52.324 | 1.947 | 26.869 | 4.388 | .013 | .012 | 8.545 | .749 |
|  | Huynh-Feldt | 52.324 | 1.963 | 26.657 | 4.388 | .013 | .012 | 8.613 | .752 |
|  | Lower-bound | 52.324 | 1.000 | 52.324 | 4.388 | .037 | .012 | 4.388 | .551 |
| Error(Stage) | Sphericity Assumed | 4412.100 | 740 | 5.962 |  |  |  |  |  |
|  | Greenhouse-Geisser | 4412.100 | 720.540 | 6.123 |  |  |  |  |  |
|  | Huynh-Feldt | 4412.100 | 726.257 | 6.075 |  |  |  |  |  |
|  | Lower-bound | 4412.100 | 370.000 | 11.925 |  |  |  |  |  |
| a. Computed using alpha = .05 | | | | | | | | | |

| **Tests of Within-Subjects Contrasts** | | | | | | | | | |
| --- | --- | --- | --- | --- | --- | --- | --- | --- | --- |
| Measure: Positive_affect | | | | | | | | | |
| Source | Stage | Type III Sum of Squares | df | Mean Square | F | Sig. | Partial Eta Squared | Noncent. Parameter | Observed Power^a^ |
| Stage | Level 2 vs. Level 1 | 17.926 | 1 | 17.926 | 1.740 | .188 | .005 | 1.740 | .260 |
|  | Level 3 vs. Level 1 | 11.002 | 1 | 11.002 | .804 | .371 | .002 | .804 | .145 |
| Stage * trees | Level 2 vs. Level 1 | 1.668 | 1 | 1.668 | .162 | .688 | .000 | .162 | .069 |
|  | Level 3 vs. Level 1 | 89.002 | 1 | 89.002 | 6.503 | .011 | .017 | 6.503 | .720 |
| Error(Stage) | Level 2 vs. Level 1 | 3811.115 | 370 | 10.300 |  |  |  |  |  |
|  | Level 3 vs. Level 1 | 5063.910 | 370 | 13.686 |  |  |  |  |  |
| a. Computed using alpha = .05 | | | | | | | | | |

| **Tests of Between-Subjects Effects** | | | | | | | | |
| --- | --- | --- | --- | --- | --- | --- | --- | --- |
| Measure: Positive_affect | | | | | | | | |
| Transformed Variable: Average | | | | | | | | |
| Source | Type III Sum of Squares | df | Mean Square | F | Sig. | Partial Eta Squared | Noncent. Parameter | Observed Power^a^ |
| Intercept | 22608.667 | 1 | 22608.667 | 1488.271 | <.001 | .801 | 1488.271 | 1.000 |
| trees | 23.720 | 1 | 23.720 | 1.561 | .212 | .004 | 1.561 | .238 |
| Error | 5620.754 | 370 | 15.191 |  |  |  |  |  |
| a. Computed using alpha = .05 | | | | | | | | |

**Estimated Marginal Means**

| **Trees * Stage** | | | | | |
| --- | --- | --- | --- | --- | --- |
| Measure: Positive_affect | | | | | |
| Binary - The participant mentioned noticing trees in the comments | Stage | Mean | Std. Error | 95% Confidence Interval | |
|  |  |  |  | Lower Bound | Upper Bound |
| no comments | 1 | 14.314 | .223 | 13.875 | 14.752 |
|  | 2 | 13.817 | .241 | 13.343 | 14.290 |
|  | 3 | 13.763 | .250 | 13.272 | 14.254 |
| noticed trees | 1 | 12.794 | .703 | 11.412 | 14.177 |
|  | 2 | 12.529 | .759 | 11.036 | 14.023 |
|  | 3 | 13.941 | .787 | 12.393 | 15.490 |
