# Supplementary material for: Does increasing biodiversity in an urban woodland setting promote positive emotional responses in humans? A stress recovery experiment using 360-degree videos of an urban woodland
Source: PLoS One. 2024 Feb 7;19(2):e0297179. doi: 10.1371/journal.pone.0297179 (PMC10849218; doi:10.1371/journal.pone.0297179)
Supplement: S1 Dataset — (ZIP) [file pone.0297179.s002.zip › Dataset and statistics output/5 - Time spent outdoors and nature connectedness.docx]

Affective responses and time spent outdoors and nature connectedness

**Time spent outdoor during childhood**

**Negative Affect
General Linear Model**

| **Notes** | | |
| --- | --- | --- |
| Output Created | | 29-MAR-2023 14:20:06 |
| Comments | |  |
| Input | Active Dataset | DataSet1 |
|  | Filter | <none> |
|  | Weight | <none> |
|  | Split File | <none> |
|  | N of Rows in Working Data File | 372 |
| Missing Value Handling | Definition of Missing | User-defined missing values are treated as missing. |
|  | Cases Used | Statistics are based on all cases with valid data for all variables in the model. |
| Syntax | | GLM na_t1 na_t2 na_t3 BY child_1 /WSFACTOR=Stage 3 Simple(1) /MEASURE=Negative_affect /METHOD=SSTYPE(3) /POSTHOC=child_1(BONFERRONI) /EMMEANS=TABLES(child_1) /PRINT=DESCRIPTIVE ETASQ OPOWER /CRITERIA=ALPHA(.05) /WSDESIGN=Stage /DESIGN=child_1. |
| Resources | Processor Time | 00:00:00.00 |
|  | Elapsed Time | 00:00:00.00 |

| **Within-Subjects Factors** | |
| --- | --- |
| Measure: Negative_affect | |
| Stage | Dependent Variable |
| 1 | na_t1 |
| 2 | na_t2 |
| 3 | na_t3 |

| **Between-Subjects Factors** | | | |
| --- | --- | --- | --- |
|  | | Value Label | N |
| Question: How much time did you spend outdoor as a child? recoded grouping lower categories together | 1 | High | 178 |
|  | 2 | Medium | 155 |
|  | 3 | Low | 39 |

| **Descriptive Statistics** | | | | |
| --- | --- | --- | --- | --- |
|  | Question: How much time did you spend outdoor as a child? recoded grouping lower categories together | Mean | Std. Deviation | N |
| Negative Affect baseline score | High | 7.8427 | 3.80573 | 178 |
|  | Medium | 7.4968 | 3.13417 | 155 |
|  | Low | 8.2564 | 3.67580 | 39 |
|  | Total | 7.7419 | 3.52550 | 372 |
| Negative Affect score after stressor | High | 9.4270 | 4.38896 | 178 |
|  | Medium | 9.3548 | 3.89929 | 155 |
|  | Low | 10.3333 | 4.60168 | 39 |
|  | Total | 9.4919 | 4.21363 | 372 |
| Negative Affect score after video | High | 6.7697 | 3.36445 | 178 |
|  | Medium | 6.3484 | 2.51126 | 155 |
|  | Low | 7.3846 | 3.76001 | 39 |
|  | Total | 6.6586 | 3.09318 | 372 |

| **Multivariate Tests**^a^ | | | | | | | | | |
| --- | --- | --- | --- | --- | --- | --- | --- | --- | --- |
| Effect | | Value | F | Hypothesis df | Error df | Sig. | Partial Eta Squared | Noncent. Parameter | Observed Power^d^ |
| Stage | Pillai's Trace | .289 | 74.860^b^ | 2.000 | 368.000 | <.001 | .289 | 149.721 | 1.000 |
|  | Wilks' Lambda | .711 | 74.860^b^ | 2.000 | 368.000 | <.001 | .289 | 149.721 | 1.000 |
|  | Hotelling's Trace | .407 | 74.860^b^ | 2.000 | 368.000 | <.001 | .289 | 149.721 | 1.000 |
|  | Roy's Largest Root | .407 | 74.860^b^ | 2.000 | 368.000 | <.001 | .289 | 149.721 | 1.000 |
| Stage * child_1 | Pillai's Trace | .003 | .322 | 4.000 | 738.000 | .863 | .002 | 1.288 | .123 |
|  | Wilks' Lambda | .997 | .321^b^ | 4.000 | 736.000 | .864 | .002 | 1.285 | .123 |
|  | Hotelling's Trace | .003 | .320 | 4.000 | 734.000 | .864 | .002 | 1.282 | .123 |
|  | Roy's Largest Root | .003 | .479^c^ | 2.000 | 369.000 | .620 | .003 | .958 | .128 |
| a. Design: Intercept + child_1 Within Subjects Design: Stage | | | | | | | | | |
| b. Exact statistic | | | | | | | | | |
| c. The statistic is an upper bound on F that yields a lower bound on the significance level. | | | | | | | | | |
| d. Computed using alpha = .05 | | | | | | | | | |

| **Mauchly's Test of Sphericity**^a^ | | | | | | | |
| --- | --- | --- | --- | --- | --- | --- | --- |
| Measure: Negative_affect | | | | | | | |
| Within Subjects Effect | Mauchly's W | Approx. Chi-Square | df | Sig. | Epsilon^b^ | | |
|  |  |  |  |  | Greenhouse-Geisser | Huynh-Feldt | Lower-bound |
| Stage | .890 | 42.929 | 2 | <.001 | .901 | .910 | .500 |
| Tests the null hypothesis that the error covariance matrix of the orthonormalized transformed dependent variables is proportional to an identity matrix. | | | | | | | |
| a. Design: Intercept + child_1 Within Subjects Design: Stage | | | | | | | |
| b. May be used to adjust the degrees of freedom for the averaged tests of significance. Corrected tests are displayed in the Tests of Within-Subjects Effects table. | | | | | | | |

| **Tests of Within-Subjects Effects** | | | | | | | | | |
| --- | --- | --- | --- | --- | --- | --- | --- | --- | --- |
| Measure: Negative_affect | | | | | | | | | |
| Source | | Type III Sum of Squares | df | Mean Square | F | Sig. | Partial Eta Squared | Noncent. Parameter | Observed Power^a^ |
| Stage | Sphericity Assumed | 1009.485 | 2 | 504.742 | 93.610 | <.001 | .202 | 187.219 | 1.000 |
|  | Greenhouse-Geisser | 1009.485 | 1.802 | 560.318 | 93.610 | <.001 | .202 | 168.650 | 1.000 |
|  | Huynh-Feldt | 1009.485 | 1.820 | 554.741 | 93.610 | <.001 | .202 | 170.345 | 1.000 |
|  | Lower-bound | 1009.485 | 1.000 | 1009.485 | 93.610 | <.001 | .202 | 93.610 | 1.000 |
| Stage * child_1 | Sphericity Assumed | 7.989 | 4 | 1.997 | .370 | .830 | .002 | 1.482 | .136 |
|  | Greenhouse-Geisser | 7.989 | 3.603 | 2.217 | .370 | .810 | .002 | 1.335 | .131 |
|  | Huynh-Feldt | 7.989 | 3.639 | 2.195 | .370 | .812 | .002 | 1.348 | .131 |
|  | Lower-bound | 7.989 | 2.000 | 3.995 | .370 | .691 | .002 | .741 | .109 |
| Error(Stage) | Sphericity Assumed | 3979.289 | 738 | 5.392 |  |  |  |  |  |
|  | Greenhouse-Geisser | 3979.289 | 664.800 | 5.986 |  |  |  |  |  |
|  | Huynh-Feldt | 3979.289 | 671.484 | 5.926 |  |  |  |  |  |
|  | Lower-bound | 3979.289 | 369.000 | 10.784 |  |  |  |  |  |
| a. Computed using alpha = .05 | | | | | | | | | |

| **Tests of Within-Subjects Contrasts** | | | | | | | | | |
| --- | --- | --- | --- | --- | --- | --- | --- | --- | --- |
| Measure: Negative_affect | | | | | | | | | |
| Source | Stage | Type III Sum of Squares | df | Mean Square | F | Sig. | Partial Eta Squared | Noncent. Parameter | Observed Power^a^ |
| Stage | Level 2 vs. Level 1 | 807.788 | 1 | 807.788 | 67.669 | <.001 | .155 | 67.669 | 1.000 |
|  | Level 3 vs. Level 1 | 253.721 | 1 | 253.721 | 34.882 | <.001 | .086 | 34.882 | 1.000 |
| Stage * child_1 | Level 2 vs. Level 1 | 10.867 | 2 | 5.434 | .455 | .635 | .002 | .910 | .124 |
|  | Level 3 vs. Level 1 | 2.420 | 2 | 1.210 | .166 | .847 | .001 | .333 | .076 |
| Error(Stage) | Level 2 vs. Level 1 | 4404.883 | 369 | 11.937 |  |  |  |  |  |
|  | Level 3 vs. Level 1 | 2683.997 | 369 | 7.274 |  |  |  |  |  |
| a. Computed using alpha = .05 | | | | | | | | | |

| **Tests of Between-Subjects Effects** | | | | | | | | |
| --- | --- | --- | --- | --- | --- | --- | --- | --- |
| Measure: Negative_affect | | | | | | | | |
| Transformed Variable: Average | | | | | | | | |
| Source | Type III Sum of Squares | df | Mean Square | F | Sig. | Partial Eta Squared | Noncent. Parameter | Observed Power^a^ |
| Intercept | 15793.500 | 1 | 15793.500 | 1637.304 | <.001 | .816 | 1637.304 | 1.000 |
| child_1 | 27.467 | 2 | 13.733 | 1.424 | .242 | .008 | 2.847 | .305 |
| Error | 3559.389 | 369 | 9.646 |  |  |  |  |  |
| a. Computed using alpha = .05 | | | | | | | | |

**Estimated Marginal Means**

| **How much Time spent outdoor as a child - recoded** | | | | |
| --- | --- | --- | --- | --- |
| Measure: Negative_affect | | | | |
| Question: How much time did you spend outdoor as a child? recoded grouping lower categories together | Mean | Std. Error | 95% Confidence Interval | |
|  |  |  | Lower Bound | Upper Bound |
| High | 8.013 | .233 | 7.555 | 8.471 |
| Medium | 7.733 | .249 | 7.243 | 8.224 |
| Low | 8.658 | .497 | 7.680 | 9.636 |

**Post Hoc Tests**

**How much Time spent outdoor as a child - recoded**

| **Multiple Comparisons** | | | | | | |
| --- | --- | --- | --- | --- | --- | --- |
| Measure: Negative_affect | | | | | | |
| Bonferroni | | | | | | |
| (I) How much Time spent outdoor as a child - recoded | (J) How much Time spent outdoor as a child - recoded | Mean Difference (I-J) | Std. Error | Sig. | 95% Confidence Interval | |
|  |  |  |  |  | Lower Bound | Upper Bound |
| High | Medium | .2798 | .34121 | 1.000 | -.5408 | 1.1004 |
|  | Low | -.6450 | .54911 | .723 | -1.9656 | .6756 |
| Medium | High | -.2798 | .34121 | 1.000 | -1.1004 | .5408 |
|  | Low | -.9248 | .55639 | .292 | -2.2629 | .4133 |
| Low | High | .6450 | .54911 | .723 | -.6756 | 1.9656 |
|  | Medium | .9248 | .55639 | .292 | -.4133 | 2.2629 |
| Based on observed means. The error term is Mean Square(Error) = 9.646. | | | | | | |

**Positive Affect**

**General Linear Model**

| **Notes** | | |
| --- | --- | --- |
| Output Created | | 29-MAR-2023 14:21:03 |
| Comments | |  |
| Input | Active Dataset | DataSet1 |
|  | Filter | <none> |
|  | Weight | <none> |
|  | Split File | <none> |
|  | N of Rows in Working Data File | 372 |
| Missing Value Handling | Definition of Missing | User-defined missing values are treated as missing. |
|  | Cases Used | Statistics are based on all cases with valid data for all variables in the model. |
| Syntax | | GLM pa_t1 pa_t2 pa_t3 BY child_1 /WSFACTOR=Stage 3 Simple(1) /MEASURE=Positive_affect /METHOD=SSTYPE(3) /POSTHOC=child_1(BONFERRONI) /EMMEANS=TABLES(child_1) /PRINT=DESCRIPTIVE ETASQ OPOWER /CRITERIA=ALPHA(.05) /WSDESIGN=Stage /DESIGN=child_1. |
| Resources | Processor Time | 00:00:00.00 |
|  | Elapsed Time | 00:00:00.02 |

| **Within-Subjects Factors** | |
| --- | --- |
| Measure: Positive_affect | |
| Stage | Dependent Variable |
| 1 | pa_t1 |
| 2 | pa_t2 |
| 3 | pa_t3 |

| **Between-Subjects Factors** | | | |
| --- | --- | --- | --- |
|  | | Value Label | N |
| Question: How much time did you spend outdoor as a child? recoded grouping lower categories together | 1 | High | 178 |
|  | 2 | Medium | 155 |
|  | 3 | Low | 39 |

| **Descriptive Statistics** | | | | |
| --- | --- | --- | --- | --- |
|  | Question: How much time did you spend outdoor as a child? recoded grouping lower categories together | Mean | Std. Deviation | N |
| Positive Affect baseline score | High | 14.5955 | 4.21517 | 178 |
|  | Medium | 13.7548 | 3.95320 | 155 |
|  | Low | 13.9231 | 4.22016 | 39 |
|  | Total | 14.1747 | 4.11743 | 372 |
| Positive Affect score after stressor | High | 14.0674 | 4.60483 | 178 |
|  | Medium | 13.4452 | 4.33221 | 155 |
|  | Low | 13.0256 | 4.00977 | 39 |
|  | Total | 13.6989 | 4.43773 | 372 |
| Positive Affect score after video | High | 14.1685 | 4.53794 | 178 |
|  | Medium | 13.3742 | 4.67398 | 155 |
|  | Low | 13.6154 | 4.41061 | 39 |
|  | Total | 13.7796 | 4.58579 | 372 |

| **Multivariate Tests**^a^ | | | | | | | | | |
| --- | --- | --- | --- | --- | --- | --- | --- | --- | --- |
| Effect | | Value | F | Hypothesis df | Error df | Sig. | Partial Eta Squared | Noncent. Parameter | Observed Power^d^ |
| Stage | Pillai's Trace | .021 | 3.873^b^ | 2.000 | 368.000 | .022 | .021 | 7.746 | .699 |
|  | Wilks' Lambda | .979 | 3.873^b^ | 2.000 | 368.000 | .022 | .021 | 7.746 | .699 |
|  | Hotelling's Trace | .021 | 3.873^b^ | 2.000 | 368.000 | .022 | .021 | 7.746 | .699 |
|  | Roy's Largest Root | .021 | 3.873^b^ | 2.000 | 368.000 | .022 | .021 | 7.746 | .699 |
| Stage * child_1 | Pillai's Trace | .005 | .419 | 4.000 | 738.000 | .795 | .002 | 1.676 | .148 |
|  | Wilks' Lambda | .995 | .418^b^ | 4.000 | 736.000 | .795 | .002 | 1.673 | .148 |
|  | Hotelling's Trace | .005 | .418 | 4.000 | 734.000 | .796 | .002 | 1.671 | .148 |
|  | Roy's Largest Root | .004 | .824^c^ | 2.000 | 369.000 | .439 | .004 | 1.649 | .191 |
| a. Design: Intercept + child_1 Within Subjects Design: Stage | | | | | | | | | |
| b. Exact statistic | | | | | | | | | |
| c. The statistic is an upper bound on F that yields a lower bound on the significance level. | | | | | | | | | |
| d. Computed using alpha = .05 | | | | | | | | | |

| **Mauchly's Test of Sphericity**^a^ | | | | | | | |
| --- | --- | --- | --- | --- | --- | --- | --- |
| Measure: Positive_affect | | | | | | | |
| Within Subjects Effect | Mauchly's W | Approx. Chi-Square | df | Sig. | Epsilon^b^ | | |
|  |  |  |  |  | Greenhouse-Geisser | Huynh-Feldt | Lower-bound |
| Stage | .969 | 11.492 | 2 | .003 | .970 | .981 | .500 |
| Tests the null hypothesis that the error covariance matrix of the orthonormalized transformed dependent variables is proportional to an identity matrix. | | | | | | | |
| a. Design: Intercept + child_1 Within Subjects Design: Stage | | | | | | | |
| b. May be used to adjust the degrees of freedom for the averaged tests of significance. Corrected tests are displayed in the Tests of Within-Subjects Effects table. | | | | | | | |

| **Tests of Within-Subjects Effects** | | | | | | | | | |
| --- | --- | --- | --- | --- | --- | --- | --- | --- | --- |
| Measure: Positive_affect | | | | | | | | | |
| Source | | Type III Sum of Squares | df | Mean Square | F | Sig. | Partial Eta Squared | Noncent. Parameter | Observed Power^a^ |
| Stage | Sphericity Assumed | 41.006 | 2 | 20.503 | 3.396 | .034 | .009 | 6.792 | .639 |
|  | Greenhouse-Geisser | 41.006 | 1.940 | 21.134 | 3.396 | .035 | .009 | 6.589 | .630 |
|  | Huynh-Feldt | 41.006 | 1.961 | 20.911 | 3.396 | .035 | .009 | 6.659 | .633 |
|  | Lower-bound | 41.006 | 1.000 | 41.006 | 3.396 | .066 | .009 | 3.396 | .452 |
| Stage * child_1 | Sphericity Assumed | 8.651 | 4 | 2.163 | .358 | .838 | .002 | 1.433 | .132 |
|  | Greenhouse-Geisser | 8.651 | 3.881 | 2.229 | .358 | .833 | .002 | 1.390 | .131 |
|  | Huynh-Feldt | 8.651 | 3.922 | 2.206 | .358 | .835 | .002 | 1.405 | .131 |
|  | Lower-bound | 8.651 | 2.000 | 4.326 | .358 | .699 | .002 | .716 | .107 |
| Error(Stage) | Sphericity Assumed | 4455.773 | 738 | 6.038 |  |  |  |  |  |
|  | Greenhouse-Geisser | 4455.773 | 715.986 | 6.223 |  |  |  |  |  |
|  | Huynh-Feldt | 4455.773 | 723.612 | 6.158 |  |  |  |  |  |
|  | Lower-bound | 4455.773 | 369.000 | 12.075 |  |  |  |  |  |
| a. Computed using alpha = .05 | | | | | | | | | |

| **Tests of Within-Subjects Contrasts** | | | | | | | | | |
| --- | --- | --- | --- | --- | --- | --- | --- | --- | --- |
| Measure: Positive_affect | | | | | | | | | |
| Source | Stage | Type III Sum of Squares | df | Mean Square | F | Sig. | Partial Eta Squared | Noncent. Parameter | Observed Power^a^ |
| Stage | Level 2 vs. Level 1 | 79.843 | 1 | 79.843 | 7.751 | .006 | .021 | 7.751 | .793 |
|  | Level 3 vs. Level 1 | 32.985 | 1 | 32.985 | 2.362 | .125 | .006 | 2.362 | .335 |
| Stage * child_1 | Level 2 vs. Level 1 | 11.697 | 2 | 5.849 | .568 | .567 | .003 | 1.136 | .144 |
|  | Level 3 vs. Level 1 | .511 | 2 | .256 | .018 | .982 | .000 | .037 | .053 |
| Error(Stage) | Level 2 vs. Level 1 | 3801.085 | 369 | 10.301 |  |  |  |  |  |
|  | Level 3 vs. Level 1 | 5152.400 | 369 | 13.963 |  |  |  |  |  |
| a. Computed using alpha = .05 | | | | | | | | | |

| **Tests of Between-Subjects Effects** | | | | | | | | |
| --- | --- | --- | --- | --- | --- | --- | --- | --- |
| Measure: Positive_affect | | | | | | | | |
| Transformed Variable: Average | | | | | | | | |
| Source | Type III Sum of Squares | df | Mean Square | F | Sig. | Partial Eta Squared | Noncent. Parameter | Observed Power^a^ |
| Intercept | 45281.975 | 1 | 45281.975 | 2988.121 | <.001 | .890 | 2988.121 | 1.000 |
| child_1 | 52.648 | 2 | 26.324 | 1.737 | .177 | .009 | 3.474 | .364 |
| Error | 5591.826 | 369 | 15.154 |  |  |  |  |  |
| a. Computed using alpha = .05 | | | | | | | | |

**Estimated Marginal Means**

| **How much Time spent outdoor as a child - recoded** | | | | |
| --- | --- | --- | --- | --- |
| Measure: Positive_affect | | | | |
| Question: How much time did you spend outdoor as a child? recoded grouping lower categories together | Mean | Std. Error | 95% Confidence Interval | |
|  |  |  | Lower Bound | Upper Bound |
| High | 14.277 | .292 | 13.703 | 14.851 |
| Medium | 13.525 | .313 | 12.910 | 14.140 |
| Low | 13.521 | .623 | 12.296 | 14.747 |

**Post Hoc Tests**

**How much Time spent outdoor as a child - recoded**

| **Multiple Comparisons** | | | | | | |
| --- | --- | --- | --- | --- | --- | --- |
| Measure: Positive_affect | | | | | | |
| Bonferroni | | | | | | |
| (I) How much Time spent outdoor as a child - recoded | (J) How much Time spent outdoor as a child - recoded | Mean Difference (I-J) | Std. Error | Sig. | 95% Confidence Interval | |
|  |  |  |  |  | Lower Bound | Upper Bound |
| High | Medium | .7524 | .42767 | .238 | -.2761 | 1.7809 |
|  | Low | .7558 | .68826 | .819 | -.8994 | 2.4110 |
| Medium | High | -.7524 | .42767 | .238 | -1.7809 | .2761 |
|  | Low | .0034 | .69737 | 1.000 | -1.6738 | 1.6805 |
| Low | High | -.7558 | .68826 | .819 | -2.4110 | .8994 |
|  | Medium | -.0034 | .69737 | 1.000 | -1.6805 | 1.6738 |
| Based on observed means. The error term is Mean Square(Error) = 15.154. | | | | | | |

**Nature connectedness**

**Negative Affect**

**General Linear Model**

| **Notes** | | |
| --- | --- | --- |
| Output Created | | 29-MAR-2023 14:22:58 |
| Comments | |  |
| Input | Active Dataset | DataSet1 |
|  | Filter | <none> |
|  | Weight | <none> |
|  | Split File | <none> |
|  | N of Rows in Working Data File | 372 |
| Missing Value Handling | Definition of Missing | User-defined missing values are treated as missing. |
|  | Cases Used | Statistics are based on all cases with valid data for all variables in the model. |
| Syntax | | GLM na_t1 na_t2 na_t3 BY ins_1 /WSFACTOR=Stage 3 Simple(1) /MEASURE=Negative_affect /METHOD=SSTYPE(3) /POSTHOC=ins_1(BONFERRONI) /EMMEANS=TABLES(ins_1) /PRINT=DESCRIPTIVE ETASQ OPOWER /CRITERIA=ALPHA(.05) /WSDESIGN=Stage /DESIGN=ins_1. |
| Resources | Processor Time | 00:00:00.00 |
|  | Elapsed Time | 00:00:00.00 |

| **Within-Subjects Factors** | |
| --- | --- |
| Measure: Negative_affect | |
| Stage | Dependent Variable |
| 1 | na_t1 |
| 2 | na_t2 |
| 3 | na_t3 |

| **Between-Subjects Factors** | | |
| --- | --- | --- |
|  | | N |
| Value of the Inclusion of Nature in Self scale | 1.00 | 20 |
|  | 2.00 | 71 |
|  | 3.00 | 54 |
|  | 4.00 | 43 |
|  | 5.00 | 103 |
|  | 6.00 | 70 |
|  | 7.00 | 11 |

| **Descriptive Statistics** | | | | |
| --- | --- | --- | --- | --- |
|  | Value of the Inclusion of Nature in Self scale | Mean | Std. Deviation | N |
| Negative Affect baseline score | 1.00 | 8.6500 | 3.89703 | 20 |
|  | 2.00 | 7.8028 | 3.47900 | 71 |
|  | 3.00 | 7.4444 | 3.40735 | 54 |
|  | 4.00 | 7.2093 | 2.85822 | 43 |
|  | 5.00 | 7.9417 | 3.41802 | 103 |
|  | 6.00 | 7.6714 | 3.73670 | 70 |
|  | 7.00 | 7.8182 | 5.68890 | 11 |
|  | Total | 7.7419 | 3.52550 | 372 |
| Negative Affect score after stressor | 1.00 | 10.3000 | 4.56647 | 20 |
|  | 2.00 | 9.7606 | 3.78518 | 71 |
|  | 3.00 | 9.7593 | 4.17059 | 54 |
|  | 4.00 | 9.1395 | 4.12069 | 43 |
|  | 5.00 | 9.5534 | 4.36503 | 103 |
|  | 6.00 | 8.9857 | 4.29186 | 70 |
|  | 7.00 | 9.0000 | 5.38516 | 11 |
|  | Total | 9.4919 | 4.21363 | 372 |
| Negative Affect score after video | 1.00 | 7.6500 | 3.97724 | 20 |
|  | 2.00 | 6.4225 | 2.62767 | 71 |
|  | 3.00 | 6.5741 | 2.66778 | 54 |
|  | 4.00 | 6.2093 | 2.05355 | 43 |
|  | 5.00 | 6.7379 | 3.02914 | 103 |
|  | 6.00 | 6.7000 | 3.66871 | 70 |
|  | 7.00 | 7.5455 | 5.52021 | 11 |
|  | Total | 6.6586 | 3.09318 | 372 |

| **Multivariate Tests**^a^ | | | | | | | | | |
| --- | --- | --- | --- | --- | --- | --- | --- | --- | --- |
| Effect | | Value | F | Hypothesis df | Error df | Sig. | Partial Eta Squared | Noncent. Parameter | Observed Power^d^ |
| Stage | Pillai's Trace | .248 | 60.083^b^ | 2.000 | 364.000 | <.001 | .248 | 120.165 | 1.000 |
|  | Wilks' Lambda | .752 | 60.083^b^ | 2.000 | 364.000 | <.001 | .248 | 120.165 | 1.000 |
|  | Hotelling's Trace | .330 | 60.083^b^ | 2.000 | 364.000 | <.001 | .248 | 120.165 | 1.000 |
|  | Roy's Largest Root | .330 | 60.083^b^ | 2.000 | 364.000 | <.001 | .248 | 120.165 | 1.000 |
| Stage * ins_1 | Pillai's Trace | .019 | .583 | 12.000 | 730.000 | .857 | .009 | 7.000 | .344 |
|  | Wilks' Lambda | .981 | .582^b^ | 12.000 | 728.000 | .857 | .010 | 6.989 | .343 |
|  | Hotelling's Trace | .019 | .581 | 12.000 | 726.000 | .858 | .010 | 6.977 | .342 |
|  | Roy's Largest Root | .014 | .861^c^ | 6.000 | 365.000 | .524 | .014 | 5.168 | .341 |
| a. Design: Intercept + ins_1 Within Subjects Design: Stage | | | | | | | | | |
| b. Exact statistic | | | | | | | | | |
| c. The statistic is an upper bound on F that yields a lower bound on the significance level. | | | | | | | | | |
| d. Computed using alpha = .05 | | | | | | | | | |

| **Mauchly's Test of Sphericity**^a^ | | | | | | | |
| --- | --- | --- | --- | --- | --- | --- | --- |
| Measure: Negative_affect | | | | | | | |
| Within Subjects Effect | Mauchly's W | Approx. Chi-Square | df | Sig. | Epsilon^b^ | | |
|  |  |  |  |  | Greenhouse-Geisser | Huynh-Feldt | Lower-bound |
| Stage | .891 | 41.823 | 2 | <.001 | .902 | .921 | .500 |
| Tests the null hypothesis that the error covariance matrix of the orthonormalized transformed dependent variables is proportional to an identity matrix. | | | | | | | |
| a. Design: Intercept + ins_1 Within Subjects Design: Stage | | | | | | | |
| b. May be used to adjust the degrees of freedom for the averaged tests of significance. Corrected tests are displayed in the Tests of Within-Subjects Effects table. | | | | | | | |

| **Tests of Within-Subjects Effects** | | | | | | | | | |
| --- | --- | --- | --- | --- | --- | --- | --- | --- | --- |
| Measure: Negative_affect | | | | | | | | | |
| Source | | Type III Sum of Squares | df | Mean Square | F | Sig. | Partial Eta Squared | Noncent. Parameter | Observed Power^a^ |
| Stage | Sphericity Assumed | 809.459 | 2 | 404.730 | 74.895 | <.001 | .170 | 149.791 | 1.000 |
|  | Greenhouse-Geisser | 809.459 | 1.804 | 448.660 | 74.895 | <.001 | .170 | 135.124 | 1.000 |
|  | Huynh-Feldt | 809.459 | 1.842 | 439.351 | 74.895 | <.001 | .170 | 137.987 | 1.000 |
|  | Lower-bound | 809.459 | 1.000 | 809.459 | 74.895 | <.001 | .170 | 74.895 | 1.000 |
| Stage * ins_1 | Sphericity Assumed | 42.410 | 12 | 3.534 | .654 | .796 | .011 | 7.848 | .388 |
|  | Greenhouse-Geisser | 42.410 | 10.825 | 3.918 | .654 | .780 | .011 | 7.080 | .364 |
|  | Huynh-Feldt | 42.410 | 11.054 | 3.837 | .654 | .783 | .011 | 7.230 | .369 |
|  | Lower-bound | 42.410 | 6.000 | 7.068 | .654 | .687 | .011 | 3.924 | .260 |
| Error(Stage) | Sphericity Assumed | 3944.868 | 730 | 5.404 |  |  |  |  |  |
|  | Greenhouse-Geisser | 3944.868 | 658.522 | 5.990 |  |  |  |  |  |
|  | Huynh-Feldt | 3944.868 | 672.475 | 5.866 |  |  |  |  |  |
|  | Lower-bound | 3944.868 | 365.000 | 10.808 |  |  |  |  |  |
| a. Computed using alpha = .05 | | | | | | | | | |

| **Tests of Within-Subjects Contrasts** | | | | | | | | | |
| --- | --- | --- | --- | --- | --- | --- | --- | --- | --- |
| Measure: Negative_affect | | | | | | | | | |
| Source | Stage | Type III Sum of Squares | df | Mean Square | F | Sig. | Partial Eta Squared | Noncent. Parameter | Observed Power^a^ |
| Stage | Level 2 vs. Level 1 | 648.003 | 1 | 648.003 | 54.061 | <.001 | .129 | 54.061 | 1.000 |
|  | Level 3 vs. Level 1 | 203.261 | 1 | 203.261 | 27.811 | <.001 | .071 | 27.811 | 1.000 |
| Stage * ins_1 | Level 2 vs. Level 1 | 40.700 | 6 | 6.783 | .566 | .757 | .009 | 3.395 | .227 |
|  | Level 3 vs. Level 1 | 18.749 | 6 | 3.125 | .428 | .861 | .007 | 2.565 | .177 |
| Error(Stage) | Level 2 vs. Level 1 | 4375.050 | 365 | 11.986 |  |  |  |  |  |
|  | Level 3 vs. Level 1 | 2667.668 | 365 | 7.309 |  |  |  |  |  |
| a. Computed using alpha = .05 | | | | | | | | | |

| **Tests of Between-Subjects Effects** | | | | | | | | |
| --- | --- | --- | --- | --- | --- | --- | --- | --- |
| Measure: Negative_affect | | | | | | | | |
| Transformed Variable: Average | | | | | | | | |
| Source | Type III Sum of Squares | df | Mean Square | F | Sig. | Partial Eta Squared | Noncent. Parameter | Observed Power^a^ |
| Intercept | 14353.782 | 1 | 14353.782 | 1472.459 | <.001 | .801 | 1472.459 | 1.000 |
| ins_1 | 28.772 | 6 | 4.795 | .492 | .814 | .008 | 2.952 | .200 |
| Error | 3558.083 | 365 | 9.748 |  |  |  |  |  |
| a. Computed using alpha = .05 | | | | | | | | |

**Estimated Marginal Means**

| **Inclusion of Nature in Self** | | | | |
| --- | --- | --- | --- | --- |
| Measure: Negative_affect | | | | |
| Value of the Inclusion of Nature in Self scale | Mean | Std. Error | 95% Confidence Interval | |
|  |  |  | Lower Bound | Upper Bound |
| 1.00 | 8.867 | .698 | 7.494 | 10.240 |
| 2.00 | 7.995 | .371 | 7.267 | 8.724 |
| 3.00 | 7.926 | .425 | 7.090 | 8.761 |
| 4.00 | 7.519 | .476 | 6.583 | 8.456 |
| 5.00 | 8.078 | .308 | 7.473 | 8.683 |
| 6.00 | 7.786 | .373 | 7.052 | 8.520 |
| 7.00 | 8.121 | .941 | 6.270 | 9.972 |

**Post Hoc Tests
 Inclusion of Nature in Self**

| **Multiple Comparisons** | | | | | | |
| --- | --- | --- | --- | --- | --- | --- |
| Measure: Negative_affect | | | | | | |
| Bonferroni | | | | | | |
| (I) Value of the Inclusion of Nature in Self scale | (J) Value of the Inclusion of Nature in Self scale | Mean Difference (I-J) | Std. Error | Sig. | 95% Confidence Interval | |
|  |  |  |  |  | Lower Bound | Upper Bound |
| 1.00 | 2.00 | .8714 | .79038 | 1.000 | -1.5468 | 3.2895 |
|  | 3.00 | .9407 | .81727 | 1.000 | -1.5597 | 3.4412 |
|  | 4.00 | 1.3473 | .84505 | 1.000 | -1.2381 | 3.9327 |
|  | 5.00 | .7890 | .76292 | 1.000 | -1.5452 | 3.1232 |
|  | 6.00 | 1.0810 | .79162 | 1.000 | -1.3410 | 3.5029 |
|  | 7.00 | .7455 | 1.17201 | 1.000 | -2.8403 | 4.3312 |
| 2.00 | 1.00 | -.8714 | .79038 | 1.000 | -3.2895 | 1.5468 |
|  | 3.00 | .0694 | .56376 | 1.000 | -1.6554 | 1.7942 |
|  | 4.00 | .4759 | .60332 | 1.000 | -1.3699 | 2.3218 |
|  | 5.00 | -.0824 | .48160 | 1.000 | -1.5558 | 1.3911 |
|  | 6.00 | .2096 | .52589 | 1.000 | -1.3994 | 1.8185 |
|  | 7.00 | -.1259 | 1.01168 | 1.000 | -3.2211 | 2.9693 |
| 3.00 | 1.00 | -.9407 | .81727 | 1.000 | -3.4412 | 1.5597 |
|  | 2.00 | -.0694 | .56376 | 1.000 | -1.7942 | 1.6554 |
|  | 4.00 | .4065 | .63814 | 1.000 | -1.5458 | 2.3589 |
|  | 5.00 | -.1517 | .52456 | 1.000 | -1.7566 | 1.4531 |
|  | 6.00 | .1402 | .56549 | 1.000 | -1.5899 | 1.8703 |
|  | 7.00 | -.1953 | 1.03282 | 1.000 | -3.3552 | 2.9646 |
| 4.00 | 1.00 | -1.3473 | .84505 | 1.000 | -3.9327 | 1.2381 |
|  | 2.00 | -.4759 | .60332 | 1.000 | -2.3218 | 1.3699 |
|  | 3.00 | -.4065 | .63814 | 1.000 | -2.3589 | 1.5458 |
|  | 5.00 | -.5583 | .56687 | 1.000 | -2.2926 | 1.1761 |
|  | 6.00 | -.2663 | .60495 | 1.000 | -2.1172 | 1.5845 |
|  | 7.00 | -.6018 | 1.05494 | 1.000 | -3.8294 | 2.6258 |
| 5.00 | 1.00 | -.7890 | .76292 | 1.000 | -3.1232 | 1.5452 |
|  | 2.00 | .0824 | .48160 | 1.000 | -1.3911 | 1.5558 |
|  | 3.00 | .1517 | .52456 | 1.000 | -1.4531 | 1.7566 |
|  | 4.00 | .5583 | .56687 | 1.000 | -1.1761 | 2.2926 |
|  | 6.00 | .2920 | .48363 | 1.000 | -1.1877 | 1.7716 |
|  | 7.00 | -.0435 | .99037 | 1.000 | -3.0736 | 2.9865 |
| 6.00 | 1.00 | -1.0810 | .79162 | 1.000 | -3.5029 | 1.3410 |
|  | 2.00 | -.2096 | .52589 | 1.000 | -1.8185 | 1.3994 |
|  | 3.00 | -.1402 | .56549 | 1.000 | -1.8703 | 1.5899 |
|  | 4.00 | .2663 | .60495 | 1.000 | -1.5845 | 2.1172 |
|  | 5.00 | -.2920 | .48363 | 1.000 | -1.7716 | 1.1877 |
|  | 7.00 | -.3355 | 1.01265 | 1.000 | -3.4337 | 2.7627 |
| 7.00 | 1.00 | -.7455 | 1.17201 | 1.000 | -4.3312 | 2.8403 |
|  | 2.00 | .1259 | 1.01168 | 1.000 | -2.9693 | 3.2211 |
|  | 3.00 | .1953 | 1.03282 | 1.000 | -2.9646 | 3.3552 |
|  | 4.00 | .6018 | 1.05494 | 1.000 | -2.6258 | 3.8294 |
|  | 5.00 | .0435 | .99037 | 1.000 | -2.9865 | 3.0736 |
|  | 6.00 | .3355 | 1.01265 | 1.000 | -2.7627 | 3.4337 |
| Based on observed means. The error term is Mean Square(Error) = 9.748. | | | | | | |

**Positive Affect**

**General Linear Model**

| **Notes** | | |
| --- | --- | --- |
| Output Created | | 29-MAR-2023 14:24:25 |
| Comments | |  |
| Input | Active Dataset | DataSet1 |
|  | Filter | <none> |
|  | Weight | <none> |
|  | Split File | <none> |
|  | N of Rows in Working Data File | 372 |
| Missing Value Handling | Definition of Missing | User-defined missing values are treated as missing. |
|  | Cases Used | Statistics are based on all cases with valid data for all variables in the model. |
| Syntax | | GLM pa_t1 pa_t2 pa_t3 BY ins_1 /WSFACTOR=Stage 3 Simple(1) /MEASURE=Positive_affect /METHOD=SSTYPE(3) /POSTHOC=ins_1(BONFERRONI) /EMMEANS=TABLES(ins_1) /PRINT=DESCRIPTIVE ETASQ OPOWER /CRITERIA=ALPHA(.05) /WSDESIGN=Stage /DESIGN=ins_1. |
| Resources | Processor Time | 00:00:00.00 |
|  | Elapsed Time | 00:00:00.00 |

| **Within-Subjects Factors** | |
| --- | --- |
| Measure: Positive_affect | |
| Stage | Dependent Variable |
| 1 | pa_t1 |
| 2 | pa_t2 |
| 3 | pa_t3 |

| **Between-Subjects Factors** | | |
| --- | --- | --- |
|  | | N |
| Value of the Inclusion of Nature in Self scale | 1.00 | 20 |
|  | 2.00 | 71 |
|  | 3.00 | 54 |
|  | 4.00 | 43 |
|  | 5.00 | 103 |
|  | 6.00 | 70 |
|  | 7.00 | 11 |

| **Descriptive Statistics** | | | | |
| --- | --- | --- | --- | --- |
|  | Value of the Inclusion of Nature in Self scale | Mean | Std. Deviation | N |
| Positive Affect baseline score | 1.00 | 12.5500 | 4.41856 | 20 |
|  | 2.00 | 13.3944 | 4.20707 | 71 |
|  | 3.00 | 13.2407 | 3.56045 | 54 |
|  | 4.00 | 14.1628 | 4.43970 | 43 |
|  | 5.00 | 14.4951 | 3.86760 | 103 |
|  | 6.00 | 15.2286 | 3.89042 | 70 |
|  | 7.00 | 17.0909 | 5.33769 | 11 |
|  | Total | 14.1747 | 4.11743 | 372 |
| Positive Affect score after stressor | 1.00 | 11.5000 | 5.21637 | 20 |
|  | 2.00 | 13.4366 | 4.49994 | 71 |
|  | 3.00 | 13.1667 | 4.13795 | 54 |
|  | 4.00 | 13.1860 | 4.01933 | 43 |
|  | 5.00 | 14.5146 | 4.18650 | 103 |
|  | 6.00 | 13.8857 | 4.49651 | 70 |
|  | 7.00 | 15.1818 | 6.16146 | 11 |
|  | Total | 13.6989 | 4.43773 | 372 |
| Positive Affect score after video | 1.00 | 10.8500 | 5.11216 | 20 |
|  | 2.00 | 13.2394 | 4.86816 | 71 |
|  | 3.00 | 13.0000 | 3.83135 | 54 |
|  | 4.00 | 14.2093 | 4.70338 | 43 |
|  | 5.00 | 14.5146 | 4.26077 | 103 |
|  | 6.00 | 14.1000 | 4.27039 | 70 |
|  | 7.00 | 15.8182 | 6.98310 | 11 |
|  | Total | 13.7796 | 4.58579 | 372 |

| **Multivariate Tests**^a^ | | | | | | | | | |
| --- | --- | --- | --- | --- | --- | --- | --- | --- | --- |
| Effect | | Value | F | Hypothesis df | Error df | Sig. | Partial Eta Squared | Noncent. Parameter | Observed Power^d^ |
| Stage | Pillai's Trace | .035 | 6.663^b^ | 2.000 | 364.000 | .001 | .035 | 13.327 | .913 |
|  | Wilks' Lambda | .965 | 6.663^b^ | 2.000 | 364.000 | .001 | .035 | 13.327 | .913 |
|  | Hotelling's Trace | .037 | 6.663^b^ | 2.000 | 364.000 | .001 | .035 | 13.327 | .913 |
|  | Roy's Largest Root | .037 | 6.663^b^ | 2.000 | 364.000 | .001 | .035 | 13.327 | .913 |
| Stage * ins_1 | Pillai's Trace | .051 | 1.583 | 12.000 | 730.000 | .092 | .025 | 18.992 | .837 |
|  | Wilks' Lambda | .950 | 1.584^b^ | 12.000 | 728.000 | .091 | .025 | 19.008 | .837 |
|  | Hotelling's Trace | .052 | 1.585 | 12.000 | 726.000 | .091 | .026 | 19.023 | .838 |
|  | Roy's Largest Root | .040 | 2.435^c^ | 6.000 | 365.000 | .025 | .038 | 14.611 | .824 |
| a. Design: Intercept + ins_1 Within Subjects Design: Stage | | | | | | | | | |
| b. Exact statistic | | | | | | | | | |
| c. The statistic is an upper bound on F that yields a lower bound on the significance level. | | | | | | | | | |
| d. Computed using alpha = .05 | | | | | | | | | |

| **Mauchly's Test of Sphericity**^a^ | | | | | | | |
| --- | --- | --- | --- | --- | --- | --- | --- |
| Measure: Positive_affect | | | | | | | |
| Within Subjects Effect | Mauchly's W | Approx. Chi-Square | df | Sig. | Epsilon^b^ | | |
|  |  |  |  |  | Greenhouse-Geisser | Huynh-Feldt | Lower-bound |
| Stage | .967 | 12.270 | 2 | .002 | .968 | .989 | .500 |
| Tests the null hypothesis that the error covariance matrix of the orthonormalized transformed dependent variables is proportional to an identity matrix. | | | | | | | |
| a. Design: Intercept + ins_1 Within Subjects Design: Stage | | | | | | | |
| b. May be used to adjust the degrees of freedom for the averaged tests of significance. Corrected tests are displayed in the Tests of Within-Subjects Effects table. | | | | | | | |

| **Tests of Within-Subjects Effects** | | | | | | | | | |
| --- | --- | --- | --- | --- | --- | --- | --- | --- | --- |
| Measure: Positive_affect | | | | | | | | | |
| Source | | Type III Sum of Squares | df | Mean Square | F | Sig. | Partial Eta Squared | Noncent. Parameter | Observed Power^a^ |
| Stage | Sphericity Assumed | 73.034 | 2 | 36.517 | 6.117 | .002 | .016 | 12.235 | .888 |
|  | Greenhouse-Geisser | 73.034 | 1.936 | 37.727 | 6.117 | .003 | .016 | 11.842 | .880 |
|  | Huynh-Feldt | 73.034 | 1.978 | 36.924 | 6.117 | .002 | .016 | 12.100 | .886 |
|  | Lower-bound | 73.034 | 1.000 | 73.034 | 6.117 | .014 | .016 | 6.117 | .694 |
| Stage * ins_1 | Sphericity Assumed | 106.758 | 12 | 8.896 | 1.490 | .122 | .024 | 17.884 | .808 |
|  | Greenhouse-Geisser | 106.758 | 11.615 | 9.191 | 1.490 | .125 | .024 | 17.310 | .798 |
|  | Huynh-Feldt | 106.758 | 11.868 | 8.996 | 1.490 | .123 | .024 | 17.687 | .805 |
|  | Lower-bound | 106.758 | 6.000 | 17.793 | 1.490 | .180 | .024 | 8.942 | .577 |
| Error(Stage) | Sphericity Assumed | 4357.667 | 730 | 5.969 |  |  |  |  |  |
|  | Greenhouse-Geisser | 4357.667 | 706.579 | 6.167 |  |  |  |  |  |
|  | Huynh-Feldt | 4357.667 | 721.959 | 6.036 |  |  |  |  |  |
|  | Lower-bound | 4357.667 | 365.000 | 11.939 |  |  |  |  |  |
| a. Computed using alpha = .05 | | | | | | | | | |

| **Tests of Within-Subjects Contrasts** | | | | | | | | | |
| --- | --- | --- | --- | --- | --- | --- | --- | --- | --- |
| Measure: Positive_affect | | | | | | | | | |
| Source | Stage | Type III Sum of Squares | df | Mean Square | F | Sig. | Partial Eta Squared | Noncent. Parameter | Observed Power^a^ |
| Stage | Level 2 vs. Level 1 | 126.814 | 1 | 126.814 | 12.622 | <.001 | .033 | 12.622 | .943 |
|  | Level 3 vs. Level 1 | 88.938 | 1 | 88.938 | 6.439 | .012 | .017 | 6.439 | .716 |
| Stage * ins_1 | Level 2 vs. Level 1 | 145.637 | 6 | 24.273 | 2.416 | .027 | .038 | 14.496 | .821 |
|  | Level 3 vs. Level 1 | 111.652 | 6 | 18.609 | 1.347 | .235 | .022 | 8.084 | .527 |
| Error(Stage) | Level 2 vs. Level 1 | 3667.145 | 365 | 10.047 |  |  |  |  |  |
|  | Level 3 vs. Level 1 | 5041.259 | 365 | 13.812 |  |  |  |  |  |
| a. Computed using alpha = .05 | | | | | | | | | |

| **Tests of Between-Subjects Effects** | | | | | | | | |
| --- | --- | --- | --- | --- | --- | --- | --- | --- |
| Measure: Positive_affect | | | | | | | | |
| Transformed Variable: Average | | | | | | | | |
| Source | Type III Sum of Squares | df | Mean Square | F | Sig. | Partial Eta Squared | Noncent. Parameter | Observed Power^a^ |
| Intercept | 42551.804 | 1 | 42551.804 | 2885.063 | <.001 | .888 | 2885.063 | 1.000 |
| ins_1 | 261.088 | 6 | 43.515 | 2.950 | .008 | .046 | 17.702 | .900 |
| Error | 5383.386 | 365 | 14.749 |  |  |  |  |  |
| a. Computed using alpha = .05 | | | | | | | | |

**Estimated Marginal Means**

| **Inclusion of Nature in Self** | | | | |
| --- | --- | --- | --- | --- |
| Measure: Positive_affect | | | | |
| Value of the Inclusion of Nature in Self scale | Mean | Std. Error | 95% Confidence Interval | |
|  |  |  | Lower Bound | Upper Bound |
| 1.00 | 11.633 | .859 | 9.945 | 13.322 |
| 2.00 | 13.357 | .456 | 12.461 | 14.253 |
| 3.00 | 13.136 | .523 | 12.108 | 14.164 |
| 4.00 | 13.853 | .586 | 12.701 | 15.004 |
| 5.00 | 14.508 | .378 | 13.764 | 15.252 |
| 6.00 | 14.405 | .459 | 13.502 | 15.307 |
| 7.00 | 16.030 | 1.158 | 13.753 | 18.307 |

**Post Hoc Tests
 Inclusion of Nature in Self**

| **Multiple Comparisons** | | | | | | |
| --- | --- | --- | --- | --- | --- | --- |
| Measure: Positive_affect | | | | | | |
| Bonferroni | | | | | | |
| (I) Value of the Inclusion of Nature in Self scale | (J) Value of the Inclusion of Nature in Self scale | Mean Difference (I-J) | Std. Error | Sig. | 95% Confidence Interval | |
|  |  |  |  |  | Lower Bound | Upper Bound |
| 1.00 | 2.00 | -1.7235 | .97221 | 1.000 | -4.6979 | 1.2510 |
|  | 3.00 | -1.5025 | 1.00528 | 1.000 | -4.5781 | 1.5732 |
|  | 4.00 | -2.2194 | 1.03945 | .702 | -5.3996 | .9608 |
|  | 5.00 | -2.8748^*^ | .93843 | .049 | -5.7459 | -.0036 |
|  | 6.00 | -2.7714 | .97373 | .098 | -5.7506 | .2077 |
|  | 7.00 | -4.3970 | 1.44162 | .052 | -8.8076 | .0137 |
| 2.00 | 1.00 | 1.7235 | .97221 | 1.000 | -1.2510 | 4.6979 |
|  | 3.00 | .2210 | .69344 | 1.000 | -1.9006 | 2.3426 |
|  | 4.00 | -.4959 | .74211 | 1.000 | -2.7664 | 1.7746 |
|  | 5.00 | -1.1513 | .59239 | 1.000 | -2.9637 | .6611 |
|  | 6.00 | -1.0480 | .64686 | 1.000 | -3.0270 | .9311 |
|  | 7.00 | -2.6735 | 1.24441 | .679 | -6.4808 | 1.1338 |
| 3.00 | 1.00 | 1.5025 | 1.00528 | 1.000 | -1.5732 | 4.5781 |
|  | 2.00 | -.2210 | .69344 | 1.000 | -2.3426 | 1.9006 |
|  | 4.00 | -.7169 | .78494 | 1.000 | -3.1184 | 1.6846 |
|  | 5.00 | -1.3723 | .64523 | .716 | -3.3464 | .6018 |
|  | 6.00 | -1.2690 | .69558 | 1.000 | -3.3971 | .8592 |
|  | 7.00 | -2.8945 | 1.27041 | .489 | -6.7813 | .9923 |
| 4.00 | 1.00 | 2.2194 | 1.03945 | .702 | -.9608 | 5.3996 |
|  | 2.00 | .4959 | .74211 | 1.000 | -1.7746 | 2.7664 |
|  | 3.00 | .7169 | .78494 | 1.000 | -1.6846 | 3.1184 |
|  | 5.00 | -.6554 | .69728 | 1.000 | -2.7887 | 1.4779 |
|  | 6.00 | -.5520 | .74411 | 1.000 | -2.8287 | 1.7246 |
|  | 7.00 | -2.1776 | 1.29762 | 1.000 | -6.1477 | 1.7925 |
| 5.00 | 1.00 | 2.8748^*^ | .93843 | .049 | .0036 | 5.7459 |
|  | 2.00 | 1.1513 | .59239 | 1.000 | -.6611 | 2.9637 |
|  | 3.00 | 1.3723 | .64523 | .716 | -.6018 | 3.3464 |
|  | 4.00 | .6554 | .69728 | 1.000 | -1.4779 | 2.7887 |
|  | 6.00 | .1033 | .59489 | 1.000 | -1.7167 | 1.9234 |
|  | 7.00 | -1.5222 | 1.21820 | 1.000 | -5.2493 | 2.2049 |
| 6.00 | 1.00 | 2.7714 | .97373 | .098 | -.2077 | 5.7506 |
|  | 2.00 | 1.0480 | .64686 | 1.000 | -.9311 | 3.0270 |
|  | 3.00 | 1.2690 | .69558 | 1.000 | -.8592 | 3.3971 |
|  | 4.00 | .5520 | .74411 | 1.000 | -1.7246 | 2.8287 |
|  | 5.00 | -.1033 | .59489 | 1.000 | -1.9234 | 1.7167 |
|  | 7.00 | -1.6255 | 1.24560 | 1.000 | -5.4365 | 2.1854 |
| 7.00 | 1.00 | 4.3970 | 1.44162 | .052 | -.0137 | 8.8076 |
|  | 2.00 | 2.6735 | 1.24441 | .679 | -1.1338 | 6.4808 |
|  | 3.00 | 2.8945 | 1.27041 | .489 | -.9923 | 6.7813 |
|  | 4.00 | 2.1776 | 1.29762 | 1.000 | -1.7925 | 6.1477 |
|  | 5.00 | 1.5222 | 1.21820 | 1.000 | -2.2049 | 5.2493 |
|  | 6.00 | 1.6255 | 1.24560 | 1.000 | -2.1854 | 5.4365 |
| Based on observed means. The error term is Mean Square(Error) = 14.749. | | | | | | |
| *. The mean difference is significant at the .05 level. | | | | | | |
