# Supplementary material for: Does increasing biodiversity in an urban woodland setting promote positive emotional responses in humans? A stress recovery experiment using 360-degree videos of an urban woodland
Source: PLoS One. 2024 Feb 7;19(2):e0297179. doi: 10.1371/journal.pone.0297179 (PMC10849218; doi:10.1371/journal.pone.0297179)
Supplement: Attachment — Submitted filename: Response to Reviewers.docx [file pone.0297179.s003.docx]

Simone Farris & Ross Cameron,

Department of Landscape Architecture,

University of Sheffield,

Floor 12 The Arts Tower,

Western Bank,

Sheffield, S10 2TN, UK.

Telephone: +44 (0)114 222 0606
sfarris1@sheffield.ac.uk

22 September 2023.

Dear Prof Banerjee,

Manuscript Number: PONE-D-23-09550

Thank you for your comments on the paper “**Does increasing biodiversity in an urban woodland setting promote positive emotional responses in humans? A Stress Recovery Experiment Using 360-degree Videos of an Urban Woodland”.** We are grateful to the reviewers for their input and guidance.

We have revised the paper based on the reviewers’ comments as outlined below.

Yours sincerely

Simone Farris & Ross Cameron

# Response to the editor’s comments

(Note. Line numbers mentioned in this response refer to the unmarked version of the revised manuscript without tracked changes)

**Please ensure that your manuscript meets PLOS ONE's style requirements, including those for file naming.**

The manuscript has now been formatted according to the journal’s style.

**In your Methods section, please provide additional information regarding the permits you obtained for the work.**

A statement concerning the permission obtained for the study has been added (Lines 150-151)

**Please include captions for your Supporting Information files at the end of your manuscript, and update any in-text citations to match accordingly.**

Captions have been added, as requested. (Lines 512-514)

**Please review your reference list to ensure that it is complete and correct.**

The reference list has been reviewed. The style is now uniform and in line with the journal’s specifications.

**Figures**

All figures’ files have been processed with PACE

# Response to the reviewers’ comments

**Comment 1). Have the authors made all data underlying the findings in their manuscript fully available? Reviewer #1: NO Reviewer #2: YES**

A dataset file is publicly available from the UK Data Service database

URL: https://reshare.ukdataservice.ac.uk/856311/

DOI: <https://dx.doi.org/10.5255/UKDA-SN-856311>

This dataset contains demographic information, computed values of the dependent variables, ratings of nature connectedness and perceived biodiversity, original textual responses from the participants and dummy variables derived by the content analysis. The dataset file is also available as part of the supporting information attached to the submission (Dataset and statistics output.zip), along with the statistical analysis outputs.

**Abstract**

**Comment 2). The participants have recorded perceptions of biodiversity, but it is not listed as one of the study's objectives.**

We thank reviewer #1 for this suggestion. The abstract now mentions *perceived* biodiversity as part of the aims (line 21). Perceptions of biodiversity were not part of the original objectives of the study, but a measure of perceptions was included to act as a control, informed by the studies cited in lines 93-99.

**Comment 3). Line 29: Change "their on" to "on their" (There are many such instances, please read and make changes)**

Changed. The text is now thoroughly proofread and all these typos have been corrected.

**Introduction**

**Comment 4). The introductory paragraph may also include about perception (with an explanation) of biodiversity, which is addressed in the later parts.**

Perceived biodiversity, and a definition of it, is now mentioned in the second paragraph after the concept of biodiversity is introduced (lines 51-54). It is more logical to place it here, as the first paragraph deals with the health benefit theories alone.

**Comment 5). Paragraphs 4 and 5 (lines 58 to 69): Consists of all the findings from the literature. Presenting these findings separately with an explanation is suggested.** **Are there any similar studies conducted using similar methods? If yes, pls cite them.**

We thank reviewer #1 for these suggestions. New summaries of previous experiments involving biodiversity have been added in Paragraphs 4 and 5. All the six studies mentioned in line 66 are now explained, making the review more systematic. Several of these studies have been mentioned for their use of similar methods. E.g. references 18 and 31 used 360-degree videos and references 29-31 have repeated measure designs. We intended to both highlight the paucity of experimental evidence and some limitations of previous studies, in particular the comparison of different locations.

**Comment 7) The description of the study conducted can be organized under the sub-heading "the present study" or similar.**

A subheading has been introduced (line 118)

**Method**

**Comment 8). Line 222 to 225: Can be part of "procedure"**

We thank reviewer #1 for this suggestion. However, we would argue that the description of the procedure already mentions the collection of demographic information (line 194). In the context of the “measures” section, lines 237-240 explain what demographic information was collected and why.

**Comment 9). Line 248 to 251: Include under the details of the measures used**

This paragraph has been moved to the “measures” section, lines 225-228.

**Results**

**Comment 10). Presenting the statistical results in a table would be easier for readers to understand.**

The results of the repeated measures ANOVAs have been summarised in a table (Table 3, line 307) to allow full comprehension of the statistics, whilst retaining the pertinent ‘p’ significant values in the text.

**Comment 11).** **Discussion can be more rigorous, specific to the Results of the study.**

The discussion has been rearranged to better discuss the three main findings: 1) Emotional responses to green space via video; 2) biodiversity levels and emotions; 3) Perceived biodiversity and affective response.

**Comment 12).** **Recommendations for future research- Not Mentioned**

We thank reviewer #1 for highlighting this. We introduced a few suggestions for future research. Lines 469-480

**Comment 13**). **Others- Table formats are not uniform**

All tables are now in the same format, in line with the journal’s guidelines

We hope these modifications clarify any outstanding points.
